# Efficacy and safety of PD-1/PD-L1 inhibitors combined with tyrosine kinase inhibitors as first-line treatment for hepatocellular carcinoma: a meta-analysis and trial sequential analysis of randomized controlled trials 

**DOI:** 10.3389/fphar.2025.1535444

**Published:** 2025-03-24

**Authors:** Peng Tang, Fei Zhou

**Affiliations:** ^1^ Department of Gastroenterology, Sichuan Provincial People’s Hospital, University of Electronic Science and Technology of China, Chengdu, China; ^2^ Department of Obstetrics and Gynaecology, Sichuan Provincial People’s Hospital, University of Electronic Science and Technology of China, Chengdu, China

**Keywords:** PD-1 inhibitor, PD-L1 inhibitor, tyrosine kinase inhibitor, combination therapy, hepatocellular carcinoma

## Abstract

**Background:**

The use of immune checkpoint inhibitors (ICIs) in treating hepatocellular carcinoma (HCC) has grown significantly. However, the therapeutic benefits of ICIs alone are notably modest. This meta-analysis assesses the efficacy and safety of using PD-1/PD-L1 inhibitors in conjunction with tyrosine kinase inhibitors (TKIs) for patients with advanced or unresectable HCC.

**Methods:**

An extensive search of the literature was performed using databases such as PubMed, Web of Science, Embase, and the Cochrane Library, capturing randomized controlled trials (RCTs) until 16 October 2024. Efficacy was measured by progression-free survival (PFS), overall survival (OS), objective response rate (ORR), and disease control rate (DCR). Safety was gauged through the occurrence of treatment-related adverse events (TRAEs). Hazard ratios (HRs) for PFS and OS, along with risk ratios (RRs) for ORR, DCR, and TRAEs, were calculated, each with 95% confidence intervals (CIs). Heterogeneity among studies was quantified using Cochran’s Q test, I^2^ statistics, and 95% prediction intervals (PIs).

**Results:**

This analysis incorporated 4 studies with a total of 2,174 patients. Treatment regimens combining PD-1/PD-L1 inhibitors with TKIs significantly improved PFS (HR = 0.694, 95% CI: 0.527–0.914; 95% PI: 0.228–2.114) and ORR (RR = 2.303, 95% CI: 1.360–3.902; 95% PI: 0.408–12.991) compared with first-line monotherapy or TKI monotherapy in the overall population. Subgroup analysis indicated that the improvements in PFS and OS were particularly significant among patients of Asian descent or those with hepatitis B virus (HBV) infection (all *p* < 0.05). While the occurrence of any grade TRAEs did not differ significantly between the two groups (RR = 1.016, 95% CI: 0.996–1.036; 95% PI: 0.941–1.097), the incidence of serious (RR = 2.068, 95% CI: 1.328–3.222; 95% PI: 0.487–8.776) and grade ≥3 TRAEs (RR = 1.287, 95% CI: 1.020–1.624; 95% PI: 0.574–2.883) increased in patients treated with the combination of PD-1/PD-L1 inhibitors and TKIs.

**Conclusion:**

This study revealed that combining PD-1/PD-L1 inhibitors with TKIs in the treatment of advanced or unresectable HCC leads to superior clinical outcomes compared to first-line monotherapy or TKIs alone, particularly in patients with HBV infection and those of Asian descent. Clinicians are advised to be vigilant regarding the potential for TRAEs in clinical settings.

## 1 Introduction

Globally, primary liver cancer poses a significant public health challenge, being the sixth most common and the third deadliest cancer type (Sung et al., 2021). Hepatocellular carcinoma (HCC) accounts for approximately 75%–85% of all primary liver cancer instances (Singal et al., 2020), with a substantial 72% of these cases diagnosed in Asia, where hepatitis B virus (HBV) infection is the predominant risk factor (Singal et al., 2020). Individuals diagnosed with HCC typically present at advanced stages; nevertheless, the introduction of targeted and immune therapies has extended their life expectancy (Llovet et al., 2021; Villanueva, 2019). The first-line systemic treatments for advanced HCC include monotherapy with the oral multitargeted tyrosine kinase inhibitors (TKIs) sorafenib and lenvatinib (Kudo et al., 2018; Llovet et al., 2008). Nonetheless, these targeted therapies have only yielded modest improvements in survival (Choi et al., 2022; De Matteis et al., 2021; Zhang et al., 2021). Moreover, it has been observed that sorafenib is less effective in patients with HBV-associated HCC compared to those without such infections (Choi et al., 2022; De Matteis et al., 2021).

In the last 5 years, immune checkpoint inhibitors (ICIs) that target the PD-1/PD-L1 pathway have been introduced as novel therapeutic options for advanced HCC (Finn et al., 2020c; Qin et al., 2020; Yau et al., 2022; Zhu et al., 2018). However, the response to ICI monotherapy remains limited to a small fraction of HCC patients (Finn et al., 2020c; Qin et al., 2020; Yau et al., 2022; Zhu et al., 2018), and it has not demonstrated a survival advantage over sorafenib in the first-line treatment context. Consequently, the integration of TKIs with PD-1 and PD-L1 ICIs has been pursued to enhance therapeutic outcomes. In the phase 1 b 116-KEYNOTE-524 study, the combination of lenvatinib and the PD-1 ICI pembrolizumab exhibited promising antitumor effects, achieving an objective response rate (ORR) of 36.0% and a median response duration of 12.6 months in patients with unresectable HCC. Additionally, these patients saw a median overall survival (OS) of 22.0 months and a median progression-free survival (PFS) of 8.6 months, alongside a manageable safety profile (Finn et al., 2020a). The phase three COSMIC-312 trial assessed the efficacy of the PD-L1 inhibitor atezolizumab combined with the multikinase inhibitor cabozantinib versus sorafenib in previously untreated patients with advanced HCC. The results indicated no significant improvement in OS for the combination therapy compared to sorafenib alone (Kelley et al., 2022). In another phase three trial, CARES-310, the efficacy of the PD-1 inhibitor camrelizumab combined with the TKI rivoceranib was evaluated as a first-line treatment. This combination significantly improved both median OS and median PFS, recording values of 22.1 months and 5.6 months, respectively, with an ORR of 25.4%, surpassing the performance of the sorafenib control group (Qin et al., 2023).

In recent times, the approach to systemic treatment of HCC has transitioned from multikinase inhibitors to regimens centered on immunotherapy that employ combination strategies (Abou-Alfa et al., 2022; Finn et al., 2020b; Finn et al., 2020c; Yau et al., 2020). Yet, when comparing the combination therapy of PD-1/PD-L1 inhibitors and TKIs with first-line monotherapy or TKI monotherapy, the outcomes have been inconsistent. In addition, previous systematic reviews and meta-analyses have primarily focused on the efficacy and safety of PD-1/PD-L1 inhibitors combined with anti-angiogenic agents for the treatment of HCC (Cao et al., 2024; Huang et al., 2023; Zhu et al., 2024). Although the pooled analysis has reported the benefits of PD-1/PD-L1 inhibitors combined with TKIs in improving OS, ORR, and disease control rate (DCR) (Liu et al., 2023), the supporting evidence is predominantly derived from prospective cohort studies, with a notable lack of evidence from randomized controlled trials (RCTs). Therefore, we undertook a meta-analysis of RCTs to comprehensively evaluate the efficacy and safety of integrating PD-1/PD-L1 inhibitors with TKIs in treating advanced or unresectable HCC. Additionally, we also examined whether specific subgroups demonstrated superior PFS and OS, aiming to identify populations that derive greater benefit from this therapeutic approach.

## 2 Materials and methods

### 2.1 Study design

Following the PRISMA guidelines, pertinent studies were screened and analyzed systematically (Page et al., 2021). Additionally, this research has been registered at the International Prospective Register of Systematic Reviews (PROSPERO) with the registration number CRD42024605243.

### 2.2 Literature retrieval

We conducted a thorough search for RCTs in several databases, including PubMed, Web of Science, Embase, and Cochrane Library, covering all publications up to 16 October 2024. The search strategy focused on two main categories: therapy-related terms such as “PD-1 inhibitors”, “PD-L1 inhibitors”, “immune checkpoint inhibitors”, “tyrosine kinase inhibitors”, “TKIs”, “pembrolizumab”, “atezolizumab”, “camrelizumab”, “nivolumab”, “sorafenib”, “lenvatinib”, and “cabozantinib”; and disease-specific terms including “hepatocellular carcinoma”, “liver cancer”, “liver neoplasms”, “hepatocarcinoma”, “HCC”, and “liver cell carcinoma”. No language constraints were imposed on the search. A comprehensive search strategy for each database is detailed in Supplementary Files S1. Further, we examined the references of all pertinent articles to find additional relevant studies.

### 2.3 Inclusion and exclusion criteria

Eligibility for inclusion in the study was determined by the following criteria: (1) RCTs; (2) Participants suffering from advanced or unresectable HCC; (3) Intervention involving a combination of PD-1/PD-L1 inhibitors and TKIs; (4) Control group treated with first-line monotherapies such as sorafenib or lenvatinib, or other TKIs administered alone or in conjunction with a placebo; (5) Reporting of outcomes including PFS, OS, ORR, DCR, any grade treatment-related adverse events (TRAEs), grade ≥3 TRAEs, or serious TRAEs. Studies were excluded if they were: (1) single-arm, non-randomized, or observational; (2) utilized monotherapy or combinations not involving PD-1/PD-L1 inhibitors with TKIs; (3) characterized by insufficient or duplicate data; (4) case reports, conference abstracts, systematic reviews, animal studies, or correspondences.

### 2.4 Data extraction and quality assessment

Two independent professionals extracted the data, gathering details such as the first author’s name, year of publication, name of the trial, phase of the study, geographical area, patient population, number of participants, ages of participants, treatment protocols for the experimental and control groups, and the duration of follow-up. The primary endpoints analyzed in the meta-analysis were PFS and OS, with secondary outcomes including ORR, DCR, any grade TRAEs, grade ≥3 TRAEs, and serious TRAEs. In cases where direct data on PFS or OS were unavailable, we used Engauge Digitizer Version 10.8 and the approach by Tierney et al. (2007) to derive these metrics from Kaplan-Meier curves (Xie et al., 2022). Quality assessment of the RCTs was independently performed by two investigators using the modified Jadad scale (Jadad et al., 1996), which evaluates RCTs on five parameters and assigns a score ranging from 0 to 7 based on aspects of randomization, allocation concealment, blinding, and the rate of dropouts/withdrawals. Trials scoring between 0 and 3 were categorized as low quality, whereas scores of 4 or above indicated high quality.

### 2.5 Statistical analysis

Statistical analyses were conducted using R software 4.3.2 and STATA Version 12.0. We calculated pooled hazard ratios (HRs) and 95% confidence intervals (CIs) for PFS and OS, in addition to pooled risk ratios RRs and 95% CIs for ORR, DCR, any grade TRAEs, grade ≥3 TRAEs, and serious TRAEs. We assessed heterogeneity using the I^2^ statistic, Cochran’s Q test, and 95% prediction intervals (PIs) (Bowden et al., 2011; IntHout et al., 2016). In the presence of significant heterogeneity (*p* < 0.1 and I^2^ > 50%), analysis proceeded under a random-effects model; otherwise, a fixed-effects model was applied (Higgins and Thompson, 2002). Subgroup analyses were performed, focusing on the stratified results for PFS and OS from the included RCTs. Sensitivity analyses were performed by sequentially excluding individual studies to assess the impact on the pooled HRs or RRs. To detect publication bias, Begg’s and Egger’s tests were utilized, indicating no significant bias with *p*-values over 0.05 (Begg and Mazumdar, 1994; Egger et al., 1997). Statistical significance was established at a two-sided *p*-value of less than 0.05.

### 2.6 Trial sequential analysis

In this meta-analysis, trial sequential analysis (TSA) was implemented to reduce the likelihood of type I and type II errors (Wetterslev et al., 2017). We conducted TSA on the PFS and OS data using STATA Version 12.0 and R software 4.3.2, employing the *a priori* information size (APIS) methodology. For binary outcomes, TSA was executed using TSA software v0.9.5.10 Beta to ascertain the required information size (RIS). When the cumulative Z-curve crossed the RIS (or APIS) boundary or the trial sequential monitoring boundary, it indicated that sufficient evidence to conclude the analysis without the need for further studies. The determination of the RIS and APIS utilized settings including a two-sided α of 0.05, a power (1-β) of 0.80, and a 15% reduction in RR.

## 3 Results

### 3.1 Study selection

**FIGURE 1:**
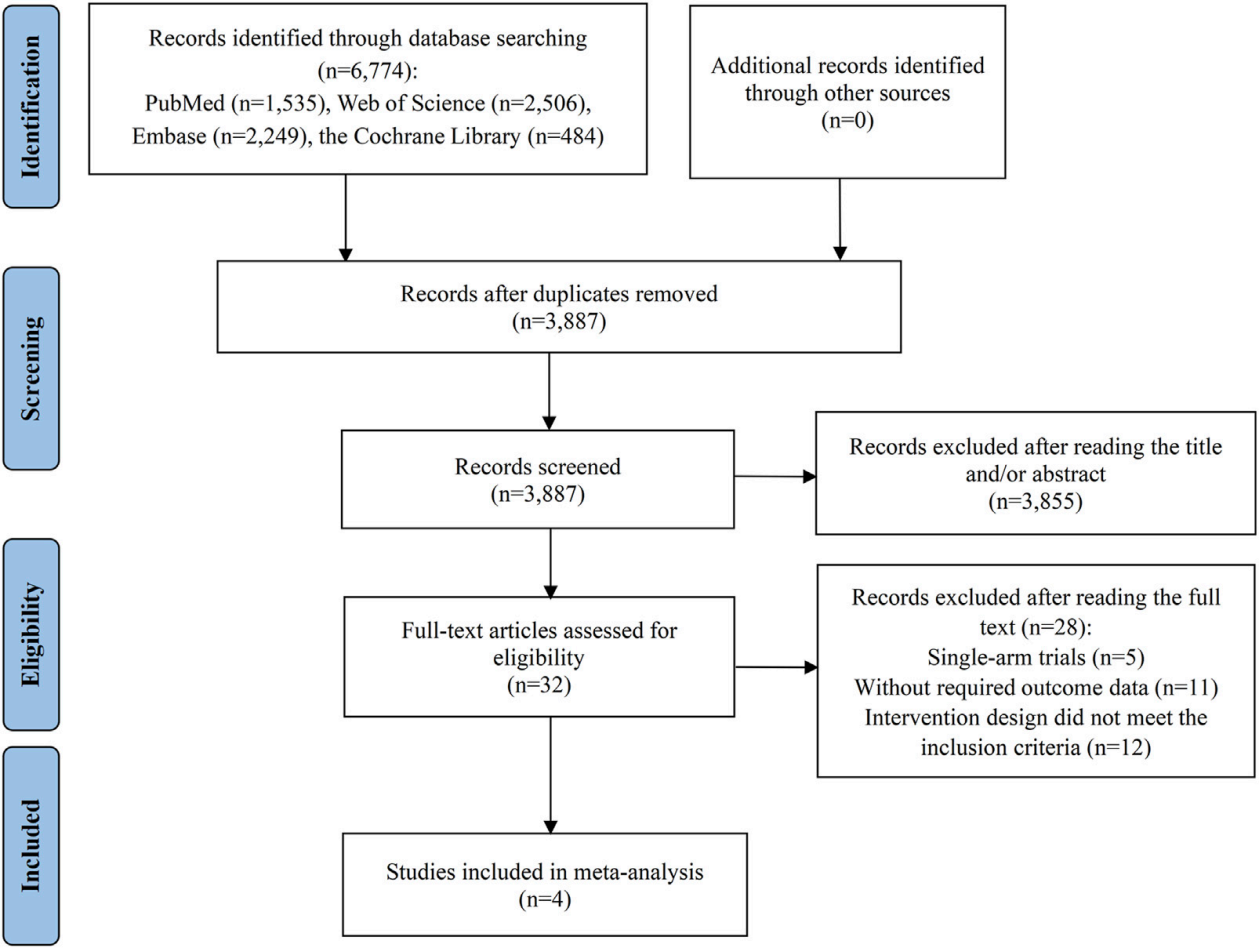
Flow diagram of the process of study selection.

Figure 1 outlines the process of literature selection used in our study. An initial search across four databases identified 6,774 potentially relevant studies. We eliminated 2,887 duplicates, then assessed the titles and abstracts of the remaining 3,887 studies. A vast majority, 3,855, were excluded for failing to meet the relevance criteria, which left 32 articles for detailed full-text evaluation to assess their suitability for inclusion. Of these, 28 studies were further excluded for various reasons: 5 were disqualified due to their single-arm trial design; 11 did not report the necessary outcome data; and 12 were rejected because their intervention group treatment regimens did not satisfy the inclusion standards. Ultimately, 4 studies qualified for inclusion in the meta-analysis (Kelley et al., 2022; Llovet et al., 2023; Qin et al., 2023; Yau et al., 2024a).

### 3.2 Characteristics and quality assessment of selected studies

Table 1 summarizes the general information, baseline patient characteristics, and therapeutic protocols. This meta-analysis covered 4 studies, including 3 Phase 3 RCTs. Notably, the COSMIC-312 trial featured two distinct control arms: one receiving sorafenib and the other cabozantinib. Yau et al. (2024b) and Kelley et al. (2022) provided differing outcomes from the COSMIC-312 trial at various follow-up intervals. We focused on extracting data from the longer follow-up periods. Additionally, outcome data not reported by Yau et al. were supplemented by findings from Kelley et al. In total, 1,099 patients with HCC were treated with a combination of PD-1/PD-L1 inhibitors and TKIs, compared to 1,075 patients who received only TKIs or TKIs plus placebo. The administered PD-1/PD-L1 inhibitors were camrelizumab, pembrolizumab, and atezolizumab, while the TKIs included sorafenib, rivoceranib, lenvatinib, and cabozantinib. These 4 studies were considered high-quality due to their stringent design (with scores ranging from 5 to 7 on the modified Jadad scale) and publication in high-impact journals. A methodological limitation noted was the lack of double-blinding in the trial design (Supplementary Files S2).

**TABLE 1:** Summary of the characteristics of included RCTs.

First author (Year)	Trial name	Study phase	Region	Patient population	Sample size (M/F)	Age (I/C, median [IQR], year)	Experimental arm	Control arm	Follow-up duration (month, median [IQR])
Qin et al. (2023)	CARES-310	Phase 3	95 study centres across 13 countries and regions	Patients (aged 18 years or older) with histopathologically or cytologically confirmed HCC; BCLC stage B or C; Not amenable to or had progressed after surgical or locoregional therapy; ECOG PS of 0 or 1	I: 227/45; C: 230/41	58 (48–66)/56 (47–64)	Camrelizumab 200 mg intravenously every 2 weeks + Rivoceranib 250 mg orally once daily (28-day cycles)	Sorafenib 400 mg orally twice daily (28-day cycles)	14.5 (9.1–18.7)
Llovet et al. (2023)	LEAP-002	Phase 3	172 global sites	Patients (aged 18 years or older) had histologically, cytologically, or radiographically confirmed HCC; ECOG PS of 0 or 1	I: 317/78; C: 327/72	66.0 (57.0–72.0)/66.0 (57.0–73.0)	Pembrolizumab 200 mg intravenously once every 3 weeks + Lenvatinib 8 mg (or 12 mg) orally once daily (up to 35 cycles)	Placebo 200 mg intravenously once every 3 weeks + Lenvatinib 8 mg (or 12 mg) orally once daily (up to 35 cycles)	32.1 (29.4–35.3)
Yau et al. (2024a)	COSMIC-312	Phase 3	178 centres in 32 countries	Patients (aged 18 years or older) had a pathological diagnosis of HCC or a radiological diagnosis of HCC in patients with cirrhosis; BCLC stage B or C; ECOG PS of 0 or 1	I: 360/72; C: 186/31(Sorafenib); 158/30 (Cabozantinib)	64 (58–70)/64 (57–71) (Sorafenib); 64 (58–71) (Cabozantinib)	Atezolizumab 1,200 mg intravenously every 3 weeks + Cabozantinib tablets 40 mg orally once daily	Sorafenib 400 mg orally twice daily	22.1 (19.3–24.8)
								Cabozantinib tablets 60 mg orally once daily	
Kelley et al. (2022)	COSMIC-312	Phase 3	178 centres in 32 countries	Patients (aged 18 years or older) had a pathological diagnosis of HCC or a radiological diagnosis of HCC in patients with cirrhosis; BCLC stage B or C; ECOG PS of 0 or 1	I: 360/72; C: 186/31(Sorafenib); 158/30 (Cabozantinib)	64 (58–70)/64 (57–71) (Sorafenib); 64 (58–71) (Cabozantinib)	Atezolizumab 1,200 mg intravenously every 3 weeks + Cabozantinib tablets 40 mg orally once daily	Sorafenib 400 mg orally twice daily	13.3 (10.5–16.0)
								Cabozantinib tablets 60 mg orally once daily	

M, male; F, female; I, intervention group; C, control group; IQR, interquartile range; HCC, hepatocellular carcinoma; BCLC, barcelona clinic liver cancer; ECOG, eastern cooperative oncology group; PS, performance status.

### 3.3 Survival outcomes

**FIGURE 2:**
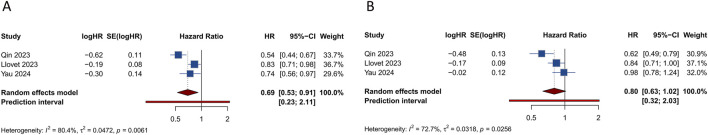
Forest plots of the survival outcomes after PD-1/PD-L1 inhibitors combined with tyrosine kinase inhibitors for hepatocellular carcinoma. **(A)** Progression-free survival **(B)** Overall survival.

Each of the 4 studies assessed PFS outcome in HCC patients. Results indicated that those treated with PD-1/PD-L1 inhibitors in conjunction with TKIs showed a significantly better PFS rate than the controls (HR = 0.694, 95% CI: 0.527–0.914; 95% PI: 0.228–2.114, I^2^ = 80.4%) (Table 2; Figure 2A). Subgroup analysis revealed that combining PD-1/PD-L1 inhibitors with TKIs significantly improved PFS in HCC patients over those receiving first-line sorafenib (HR = 0.624, 95% CI: 0.459–0.849; 95% PI: 0.029–13.563, I^2^ = 68.6%) or TKIs with placebo (HR = 0.830, 95% CI: 0.707–0.975) (Table 2; Supplementary Figure S1). Additionally, we obtained stratified analysis outcomes for PFS from the included studies based on factors including age, sex, region, race, Eastern Cooperative Oncology Group (ECOG) performance status, Barcelona Clinic Liver Cancer (BCLC) stage, baseline alpha-fetoprotein, disease aetiology, macrovascular invasion. These stratified findings were consolidated to form detailed subgroup analyses of PFS, as outlined in Table 2 and Supplementary Figures S2-S10. Significantly, the therapeutic regimen combining PD-1/PD-L1 inhibitors with TKIs was particularly effective in enhancing PFS among male patients, those from Asian regions, of Asian ethnicity, with an ECOG performance status of 0, diagnosed with BCLC stage C, and those whose disease etiology was related to hepatitis B or C virus, as well as those presenting with macrovascular invasion (all *p* < 0.05). In contrast, among females, individuals from regions other than Asia, Caucasians, patients with an ECOG performance status of 1, BCLC stage B, non-viral disease etiology, and without macrovascular invasion, the improvement in PFS was not significant when compared to controls (all *p* > 0.05).

**TABLE 2:** Pooled effect and subgroup analysis of the primary outcomes of PD-1/PD-L1 inhibitors combined with tyrosine kinase inhibitors as first-line treatment for hepatocellular carcinoma.

Outcomes and subgroups	Number of studies	Meta-analysis				Heterogeneity	
		HR	95% CI	*p* value	95% PI	I^2^, tau^2^	*p* value
PFS							
Overall	3	0.694	0.527–0.914	0.009	0.228–2.114	80.4%, 0.0472	0.006
Subgrouped by control groups							
PD-1/PD-L1 inhibitors + TKIs vs Sorafenib alone	2	0.624	0.459–0.849	0.003	0.029–13.563	68.6%, 0.0341	0.074
PD-1/PD-L1 inhibitors + TKIs vs TKIs + Placebo	1	0.830	0.707–0.975	0.023			
Age							
<65 years	2	0.696	0.527–0.918	0.010	0.050–9.594	56.4%, 0.0227	0.130
≥65 years	3	0.651	0.508–0.834	0.001	0.237–1.816	36.4%, 0.0294	0.208
Sex							
Male	2	0.634	0.543–0.741	<0.001	0.090–4.471	47.8%, 0.0115	0.166
Female	2	0.817	0.400–1.666	0.577	0.001–1,058.553	70.2%, 0.1858	0.067
Region							
Asia	2	0.562	0.466–0.679	<0.001	0.166–1.907	0%, 0	0.808
Other	2	0.750	0.483–1.162	0.198	0.010–54.510	59.4%, 0.0637	0.117
Race							
Asian	2	0.567	0.469–0.687	<0.001	0.165–1.957	0%, 0	0.936
White	2	0.763	0.489–1.189	0.232	0.011–53.509	55.3%, 0.0605	0.135
ECOG performance status							
0	2	0.697	0.573–0.847	<0.001	0.197–2.467	0%, 0	0.472
1	2	0.663	0.418–1.052	0.081	0.006–79.414	77.3%, 0.0863	0.036
BCLC stage							
Stage B	2	0.934	0.687–1.270	0.663	0.127–6.862	0%, 0	0.323
Stage C	2	0.590	0.501–0.694	<0.001	0.118–2.969	30.4%, 0.0061	0.231
Baseline alpha-fetoprotein (ng/mL)							
<400	2	0.749	0.625–0.897	0.002	0.080–7.022	46.9%, 0.0150	0.170
≥400	2	0.464	0.362–0.594	<0.001	0.042–5.114	29.2%, 0.0132	0.235
Disease aetiology							
Hepatitis B virus	2	0.554	0.456–0.674	<0.001	0.156–1.970	0%, 0	0.672
Hepatitis C virus	2	0.709	0.503–0.998	0.049	0.015–31.177	26.2%, 0.0365	0.245
Non-viral	2	0.764	0.431–1.353	0.356	0.002–252.109	71.3%, 0.1231	0.062
Macrovascular invasion							
Yes	2	0.536	0.400–0.718	<0.001	0.081–3.569	0%, 0	0.643
No	2	0.714	0.457–1.118	0.141	0.006–85.298	85.8%, 0.0895	0.008
OS							
Overall	3	0.804	0.634–1.019	0.071	0.318–2.032	72.7%, 0.0318	0.026
Subgrouped by control groups							
PD-1/PD-L1 inhibitors + TKIs vs Sorafenib alone	2	0.781	0.499–1.223	0.280	0.007–94.385	85.9%, 0.0900	0.008
PD-1/PD-L1 inhibitors + TKIs vs TKIs + Placebo	1	0.840	0.708–0.997	0.046			
Age							
<65 years	3	0.866	0.638–1.175	0.356	0.264–2.846	71.7%, 0.0522	0.029
≥65 years	4	0.797	0.670–0.947	0.010	0.602–1.054	0%, 0	0.445
Sex							
Male	3	0.787	0.606–1.023	0.073	0.280–2.214	74.8%, 0.0399	0.019
Female	3	1.049	0.785–1.403	0.746	0.554–1.986	0%, 0	0.773
Region							
Asia	2	0.688	0.550–0.860	0.001	0.162–2.924	0%, 0	0.548
Other	2	0.834	0.420–1.657	0.604	0.001–1,053.612	76.4%, 0.1931	0.040
Race							
Asian	2	0.666	0.533–0.834	<0.001	0.156–2.849	0%, 0	0.903
White	2	0.901	0.375–2.162	0.815	-	84.3%, 0.3398	0.012
ECOG performance status							
0	3	0.882	0.666–1.167	0.378	0.307–2.528	65.3%, 0.0395	0.056
1	3	0.741	0.619–0.887	0.001	0.470–1.169	7.7%, 0.0021	0.339
BCLC stage							
Stage B	3	0.999	0.770–1.297	0.995	0.428–2.298	19.2%, 0.0145	0.290
Stage C	3	0.778	0.633–0.956	0.017	0.369–1.640	57.4%, 0.0190	0.096
Child-Pugh classification							
A5	2	0.910	0.781–1.060	0.227	0.232–3.615	22.2%, 0.0037	0.257
A6	2	0.814	0.594–1.115	0.199	0.106–6.266	0%, 0	0.782
Baseline alpha-fetoprotein (ng/mL)							
<400	3	0.917	0.665–1.264	0.596	0.257–3.273	75.9%, 0.0606	0.016
≥400	3	0.636	0.527–0.768	<0.001	0.421–0.961	0%, 0	0.887
Disease aetiology							
Hepatitis B virus	3	0.693	0.584–0.822	<0.001	0.476–1.009	0%, 0	0.708
Hepatitis C virus	3	0.930	0.725–1.193	0.569	0.263–3.015	44.2%, 0.0454	0.167
Non-viral	3	0.962	0.785–1.179	0.709	0.321–2.885	49.9%, 0.0388	0.136
Extrahepatic metastasis							
Yes	2	0.673	0.488–0.930	0.016	0.027–16.911	67.9%, 0.0373	0.078
No	2	0.930	0.732–1.180	0.549	0.198–4.374	0%, 0	0.590
Macrovascular invasion							
Yes	3	0.726	0.570–0.925	0.010	0.427–1.235	0%, 0	0.607
No	3	0.864	0.673–1.110	0.253	0.332–2.253	68.5%, 0.0333	0.042

PFS, progression-free survival; TKIs, tyrosine kinase inhibitors; ECOG, eastern cooperative oncology group; BCLC, barcelona clinic liver cancer; OS, overall survival.

4 studies investigated the effects of combining PD-1/PD-L1 inhibitors with TKIs on OS in HCC patients. The combined data indicated that the addition of PD-1/PD-L1 inhibitors to TKI regimens did not significantly enhance OS relative to the control group (HR = 0.804, 95% CI: 0.634–1.019; 95% PI: 0.318–2.032, I^2^ = 72.7%) (Table 2; Figure 2B). Subgroup analyses were performed according to the specific TKIs used in the control groups. These analyses demonstrated that the co-administration of PD-1/PD-L1 inhibitors with TKIs did not yield an OS benefit over the use of sorafenib alone (HR = 0.781, 95% CI: 0.499–1.223; 95% PI: 0.007–94.385, I^2^ = 85.9%). However, a notable improvement in OS was observed with the combination of PD-1/PD-L1 inhibitors and TKIs compared to TKIs plus placebo, though this result was based on a single study (HR = 0.840, 95% CI: 0.708–0.997) (Table 2; Supplementary Figure S11). Detailed results of the OS subgroup analysis, stratified by the data from the included studies, are provided in Table 2 and Supplementary Figures S12-S22.

### 3.4 Tumor responses

**FIGURE 3:**
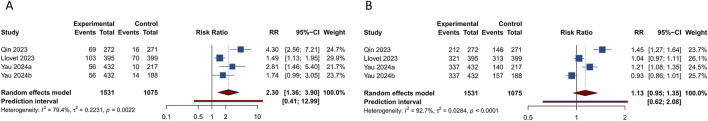
Forest plots of tumor responses after PD-1/PD-L1 inhibitors combined with tyrosine kinase inhibitors for hepatocellular carcinoma. **(A)** Objective response rate **(B)** Disease control rate.

Figure 3 illustrates tumor responses, including ORR and DCR, as forest plots. These metrics were each evaluated in 4 studies. The comprehensive assessment revealed that the ORR for the combination of PD-1/PD-L1 inhibitors with TKIs in treating HCC was significantly superior to that observed in the control group (RR = 2.303, 95% CI: 1.360–3.902; 95% PI: 0.408–12.991, I^2^ = 79.4%). Subgroup analyses further indicated that this combination therapy achieved a higher ORR compared to either sorafenib alone or TKIs alone (or combined with placebo) (all *p* < 0.05). Nonetheless, analyses showed no significant differences in DCR between patients receiving the combination therapy and those in the control groups (RR = 1.134, 95% CI: 0.955–1.347; 95% PI: 0.619–2.076, I^2^ = 92.7%). Further subgroup analysis suggested an improved DCR with the PD-1/PD-L1 inhibitors and TKIs combination compared to sorafenib alone (RR = 1.319, 95% CI: 1.106–1.573; 95% PI: 0.214–8.143, I^2^ = 77.1%), but not when compared to TKIs alone (or with placebo) (RR = 0.986, 95% CI: 0.891–1.091; 95% PI: 0.352–2.759, I^2^ = 72.3%) (Table 3; Supplementary Figures S23, S24).

**TABLE 3:** Pooled effect of the secondary outcomes of PD-1/PD-L1 inhibitors combined with tyrosine kinase inhibitors as first-line treatment for hepatocellular carcinoma.

Outcomes	Number of studies	Meta-analysis				Heterogeneity	
		RR	95% CI	*p* value	95% PI	I^2^, tau^2^	*p* value
ORR							
Overall	4	2.303	1.360–3.902	0.002	0.408–12.991	79.4%, 0.2231	0.002
PD-1/PD-L1 inhibitors + TKIs vs Sorafenib alone	2	3.624	2.421–5.424	<0.001	0.264–50.526	0%, 0	0.319
PD-1/PD-L1 inhibitors + TKIs vs TKIs alone (or plus placebo)	2	1.542	1.208–1.969	0.001	0.317–7.405	0%, 0	0.616
DCR							
Overall	4	1.134	0.955–1.347	0.152	0.619–2.076	92.7%, 0.0284	<0.001
PD-1/PD-L1 inhibitors + TKIs vs Sorafenib alone	2	1.319	1.106–1.573	0.002	0.214–8.143	77.1%, 0.0125	0.037
PD-1/PD-L1 inhibitors + TKIs vs TKIs alone (or plus placebo)	2	0.986	0.891–1.091	0.782	0.352–2.759	72.3%, 0.0039	0.058
Any grade TRAEs							
Overall	4	1.016	0.996–1.036	0.114	0.941–1.097	49.6%, 0.0004	0.114
PD-1/PD-L1 inhibitors + TKIs vs Sorafenib alone	2	1.038	1.006–1.072	0.021	0.853–1.273	0%, 0	0.384
PD-1/PD-L1 inhibitors + TKIs vs TKIs alone (or plus placebo)	2	0.998	0.974–1.022	0.857	0.856–1.168	0.9%, <0.0001	0.315
Hypertension	4	1.102	0.767–1.584	0.599	0.316–3.845	90.8%, 0.1199	<0.001
Aspartate aminotransferase increased	4	1.501	1.114–2.022	0.008	0.580–3.882	74.2%, 0.0660	0.009
Proteinuria	4	1.210	0.756–1.935	0.427	0.255–5.732	86.9%, 0.1815	<0.001
Alanine aminotransferase increased	4	1.623	1.097–2.402	0.015	0.443–5.947	82.5%, 0.1265	0.001
Platelet count decreased	4	1.250	1.070–1.460	0.005	0.692–2.278	41.9%, 0.0216	0.160
Blood bilirubin increased	4	1.269	1.071–1.504	0.006	0.695–2.221	34.8%, 0.0193	0.204
Palmar-plantar erythrodysaesthesia syndrome	4	0.886	0.690–1.137	0.341	0.379–2.072	85.2%, 0.0551	<0.001
Diarrhoea	4	0.976	0.825–1.153	0.772	0.582–1.635	65.7%, 0.0190	0.033
Hypothyroidism	4	2.282	1.105–4.709	0.026	0.189–27.547	92.2%, 0.4760	<0.001
Fatigue	4	1.328	0.854–2.065	0.208	0.298–5.927	85.4%, 0.1702	<0.001
Rash	4	0.997	0.668–1.487	0.987	0.274–3.631	74.2%, 0.1233	0.009
Decreased appetite	4	1.080	0.772–1.511	0.653	0.359–3.254	78.8%, 0.0907	0.003
Weight decreased	4	0.914	0.591–1.413	0.686	0.218–3.833	78.2%, 0.1535	0.003
Asthenia	4	1.148	0.930–1.418	0.199	0.548–2.488	42.0%, 0.0353	0.160
Nausea	4	1.153	0.789–1.684	0.463	0.357–3.721	67.2%, 0.0982	0.027
Lipase increased	4	1.950	1.407–2.702	<0.001	1.138–3.272	0%, 0	0.936
Grade ≥ 3 TRAEs							
Overall	4	1.287	1.020–1.624	0.033	0.574–2.883	90.4%, 0.0502	<0.001
PD-1/PD-L1 inhibitors + TKIs vs Sorafenib alone	2	1.590	1.419–1.780	<0.001	0.779–3.181	0%, 0	0.543
PD-1/PD-L1 inhibitors + TKIs vs TKIs alone (or plus placebo)	2	1.057	0.965–1.158	0.233	0.587–1.908	0%, 0	0.431
Hypertension	4	1.179	0.639–2.176	0.599	0.145–9.604	88.5%, 0.3367	<0.001
Aspartate aminotransferase increased	4	2.177	1.564–3.030	<0.001	0.798–5.661	29.9%, 0.0517	0.233
Proteinuria	4	1.854	1.175–2.926	0.008	0.209–25.115	47.0%, 0.3555	0.129
Alanine aminotransferase increased	4	2.198	1.154–4.187	0.017	0.309–15.639	63.8%, 0.2721	0.041
Platelet count decreased	4	1.890	0.632–5.649	0.255	0.054–65.684	77.0%, 0.9310	0.005
Blood bilirubin increased	4	2.706	1.556–4.705	<0.001	0.335–21.454	34.7%, 0.2461	0.204
Palmar-plantar erythrodysaesthesia syndrome	4	0.952	0.730–1.241	0.717	0.613–1.455	0%, 0	0.541
Diarrhoea	4	1.075	0.474–2.434	0.863	0.083–13.959	71.3%, 0.4752	0.015
Fatigue	4	1.334	0.620–2.868	0.461	0.158–11.295	50.9%, 0.2980	0.106
Rash	4	1.943	0.800–4.717	0.142	0.411–7.936	0%, 0	0.774
Decreased appetite	4	0.565	0.321–0.992	0.047	0.219–1.438	0%, 0	0.446
Weight decreased	4	0.788	0.466–1.333	0.374	0.307–1.920	1.8%, 0.0056	0.383
Asthenia	4	1.496	0.891–2.511	0.128	0.592–3.315	0%, 0	0.408
Nausea	4	0.334	0.113–0.986	0.047	0.004–26.811	21.9%, 0.4533	0.278
Lipase increased	4	1.519	0.919–2.510	0.103	0.655–3.414	0%, 0	0.671
Serious TRAEs							
Overall	4	2.068	1.328–3.222	0.001	0.487–8.776	77.4%, 0.1551	0.004
PD-1/PD-L1 inhibitors + TKIs vs Sorafenib alone	2	3.092	1.803–5.303	<0.001	0.020–483.941	54.3%, 0.0824	0.139
PD-1/PD-L1 inhibitors + TKIs vs TKIs alone (or plus placebo)	2	1.490	1.179–1.883	0.001	0.328–6.791	0%, 0	0.796

ORR, objective response rate; TKIs, tyrosine kinase inhibitors; DCR, disease control rate; TRAEs, treatment-related adverse events.

### 3.5 Treatment-related adverse events

**FIGURE 4:**
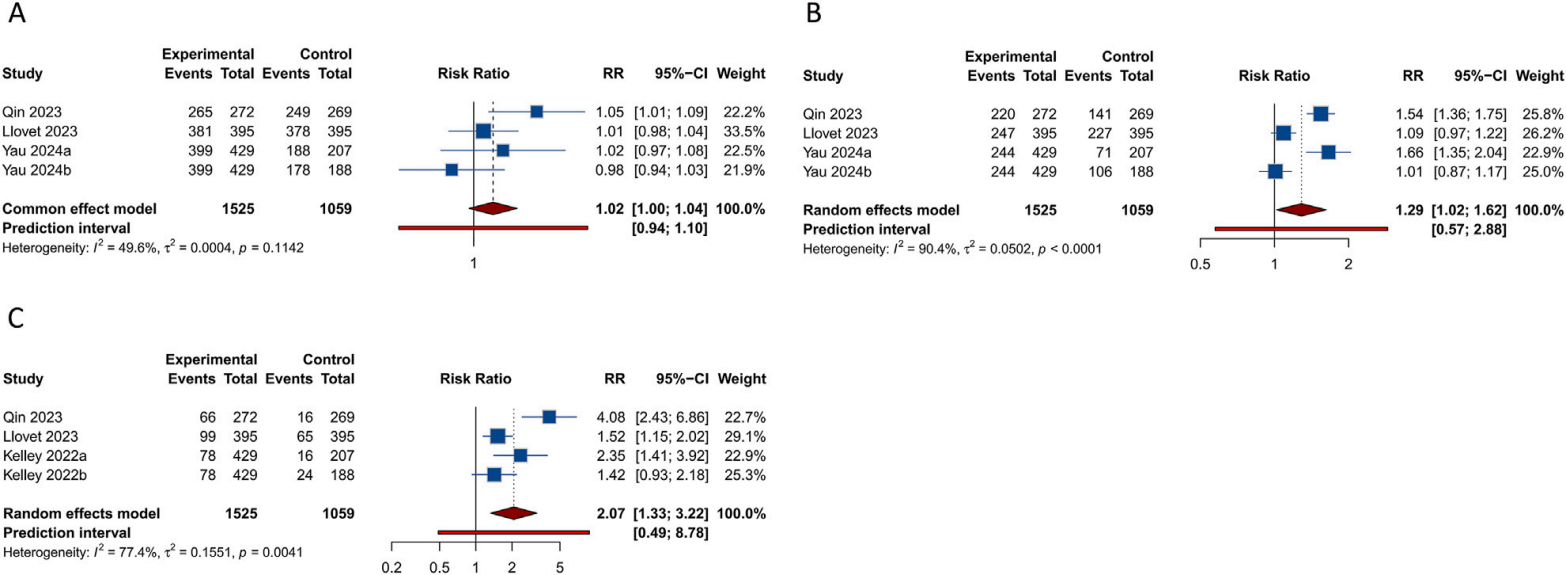
Forest plots of the safety outcomes after PD-1/PD-L1 inhibitors combined with tyrosine kinase inhibitors for hepatocellular carcinoma. **(A)** Any grade treatment-related adverse events (TRAEs) **(B)** Grade ≥3 TRAEs **(C)** Serious TRAEs.

4 studies evaluated the occurrence of any grade TRAEs within experimental and control groups. The overall analysis indicated that there was no significant difference in the incidence of any grade TRAEs between the cohort treated with PD-1/PD-L1 inhibitors combined with TKIs and the control group (RR = 1.016, 95% CI: 0.996–1.036; 95% PI: 0.941–1.097, I^2^ = 49.6%) (Figure 4A). Nevertheless, subgroup analyses demonstrated that this combination therapy led to a higher risk of any grade TRAEs compared to treatment with sorafenib alone (RR = 1.038, 95% CI: 1.006–1.072; 95% PI: 0.853–1.273, I^2^ = 0%). Specifically, the combination therapy was associated with significantly higher incidences of increased aspartate aminotransferase (AST), increased alanine aminotransferase (ALT), decreased platelet count, increased blood bilirubin, hypothyroidism, and increased lipase compared with the control (all *p* < 0.05). Conversely, there were no notable differences in the incidence of hypertension, proteinuria, palmar-plantar erythrodysesthesia syndrome, diarrhea, fatigue, rash, reduced appetite, weight loss, asthenia, and nausea between the experimental and control groups (all *p* > 0.05) (Table 3; Supplementary Figures S25-S28).

Analysis from 4 studies revealed a significantly elevated occurrence of grade ≥3 TRAEs in patients receiving a combination of PD-1/PD-L1 inhibitors and TKIs compared to those in the control group (RR = 1.287, 95% CI: 1.020–1.624; 95% PI: 0.574–2.883, I^2^ = 90.4%) (Figure 4B). Subsequent subgroup analyses further confirmed that this combination therapy increased the risk of grade ≥3 TRAEs relative to sorafenib monotherapy (RR = 1.590, 95% CI: 1.419–1.780; 95% PI: 0.779–3.181, I^2^ = 0%). In particular, treatment with the combination therapy significantly increased the occurrences of elevated AST, proteinuria, elevated ALT, and increased blood bilirubin, while simultaneously reducing the incidence of decreased appetite and nausea relative to the control group (all *p* < 0.05). However, no significant differences were observed in the rates of grade ≥3 hypertension, reduced platelet count, palmar-plantar erythrodysesthesia syndrome, diarrhea, fatigue, rash, weight loss, asthenia, and increased lipase between the experimental and control cohorts (all *p* > 0.05) (Table 3; Supplementary Figures S29-S32).

4 investigations evaluated the incidence of serious TRAEs. The comprehensive analysis indicated that the regimen combining PD-1/PD-L1 inhibitors with TKIs was associated with an increased occurrence of serious TRAEs compared to the control group (RR = 2.068, 95% CI: 1.328–3.222; 95% PI: 0.487–8.776, I^2^ = 77.4%) (Figure 4C). Moreover, this increase in risk was also observed when the combination therapy was compared to either sorafenib monotherapy or TKIs alone (or in conjunction with placebo) (all *p* < 0.05) (Table 3; Supplementary Figure S33).

### 3.6 Sensitivity analysis and publication bias

In this study, a leave-one-out sensitivity analysis was carried out to assess the influence of each individual study on the overall pooled HRs and RRs. Given the limited number of studies included, the sensitivity analysis revealed that the exclusion of individual study could potentially affect the overall results (Supplementary Figure S34). To further evaluate publication bias, we applied both funnel plots and Begg’s and Egger’s tests. These methods collectively found no indication of publication bias in the outcomes related to efficacy and safety (all *p* > 0.05). The associated funnel plots can be found in Supplementary Figure S35.

### 3.7 Trial sequential analysis results

**FIGURE 5:**
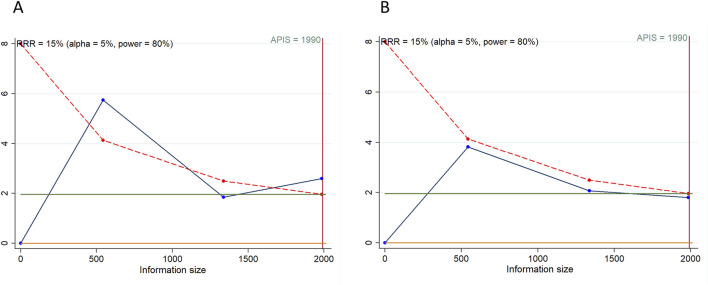
Trial sequential analysis of PD-1/PD-L1 inhibitors combined with tyrosine kinase inhibitors for hepatocellular carcinoma. **(A)** Progression-free survival **(B)** Overall survival. Red inward-sloping line to the left represents trial sequential monitoring boundary. Blue line represents evolution of cumulative Z-score. Horizontal green lines represent the conventional boundaries for statistical significance. Heterogeneity-adjusted required information size to demonstrate or reject 15% relative risk (*a priori* estimate) of mortality risk (with alpha of 5% and beta of 20%) is 1,990 patients for PFS and OS (vertical red line). Cumulative Z-curve crossing the trial sequential monitoring boundary or the APIS boundary provides firm evidence of effect.

**FIGURE 6:**
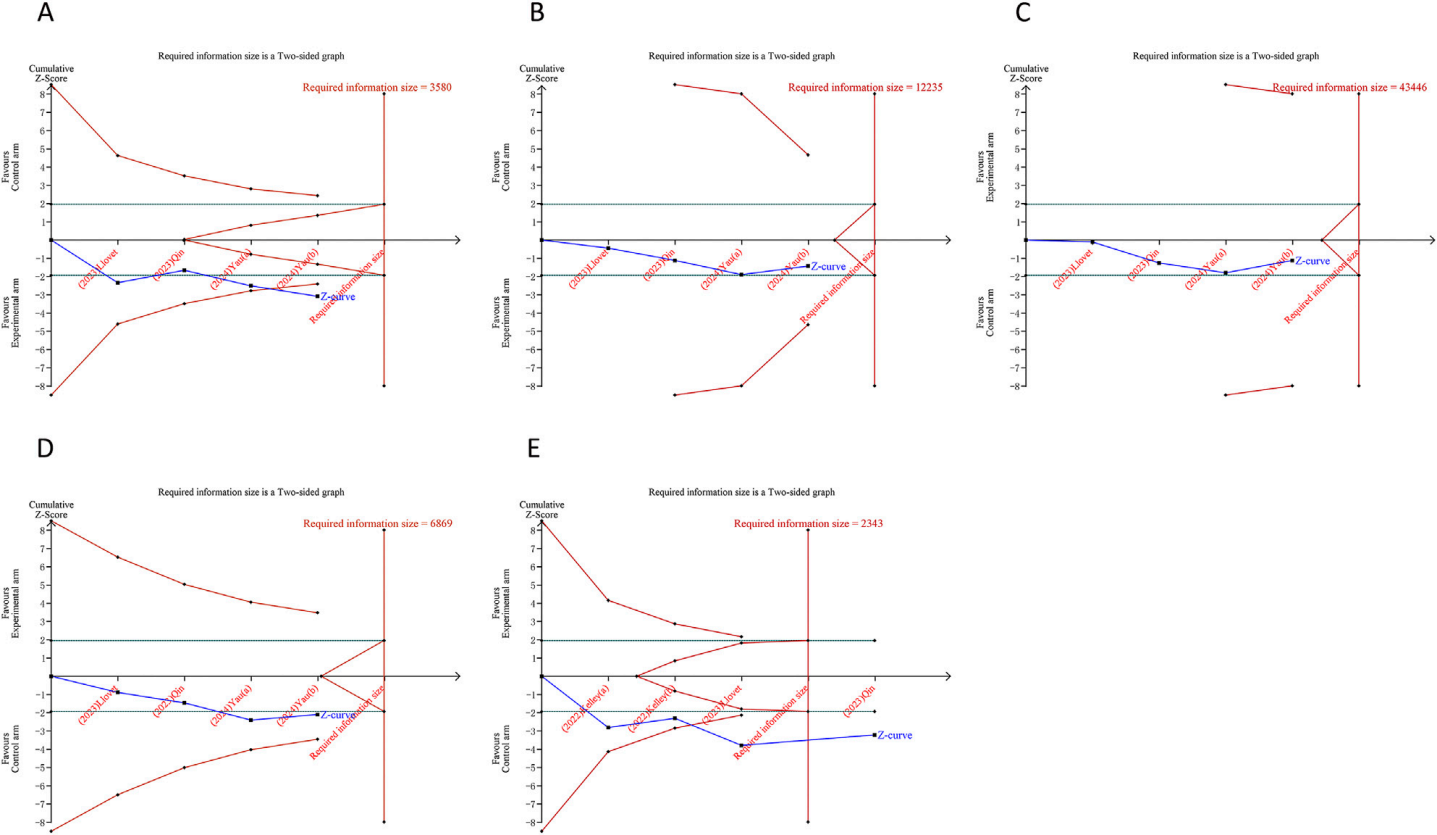
Trial sequential analysis of PD-1/PD-L1 inhibitors combined with tyrosine kinase inhibitors for hepatocellular carcinoma. **(A)** Objective response rate **(B)** Disease control rate **(C)** Any grade treatment-related adverse events (TRAEs) **(D)** Grade ≥3 TRAEs **(E)** Serious TRAEs. Uppermost and lowermost red curves represent trial sequential monitoring boundary lines for benefit and harm, respectively. Inner red lines represent the futility boundary. Blue line represents evolution of cumulative Z-score. Horizontal green lines represent the conventional boundaries for statistical significance. Cumulative Z-curve crossing the trial sequential monitoring boundary or the RIS boundary provides firm evidence of effect.

In the TSA for PFS and OS, we calculated an APIS of 1,990. It was observed that the cumulative Z-curves for PFS, ORR, and serious TRAEs crossed the trial sequential monitoring boundary, though they did not exceed the RIS boundary. This suggests the potential for drawing robust conclusions from these parameters. However, the cumulative Z-curves for OS, DCR, any grade TRAEs, and grade ≥3 TRAEs did not breach either the RIS threshold or the trial sequential monitoring boundary, indicating that these findings remain inconclusive and potentially subject to false positives (Figures 5, 6).

## 4 Discussion

In the treatment of advanced HCC, single-agent ICIs have demonstrated ORRs of 15%–20%, generally without notable improvements in OS. Additionally, intrinsic resistance to ICIs occurs in approximately 30% of HCC cases (Rimassa et al., 2023). With no predictive biomarkers available to determine which patients would most benefit from immunotherapy, researchers have shifted focus to evaluate combination therapies that might be effective in a wider range of patients. Among these, the combination of PD-1/PD-L1 inhibitors with TKIs has emerged as a particularly promising strategy for advanced HCC. Our meta-analysis, which pooled data from RCTs, found that this combination therapy significantly improved PFS and ORR when compared to either first-line monotherapy or TKI monotherapy. However, it also raised the incidence of grade ≥3 and serious TRAEs. Additionally, the combined regimen of PD-1/PD-L1 inhibitors and TKIs did not significantly impact OS, DCR, or the occurrence of any grade TRAEs.

The reasons for the discrepancy between PFS and OS in our analysis remain uncertain. Numerous oncology studies have demonstrated a weak association between PFS and OS, with one proposed explanation being that OS may be adversely impacted by reduced treatment duration due to toxicity (Merino et al., 2023). Furthermore, the combination of PD-1/PD-L1 inhibitors with TKIs is associated with an increased incidence of immune-related AEs, which may necessitate dose reductions, interruptions, or discontinuation of therapy (Yau et al., 2024b), thereby potentially diminishing overall therapeutic efficacy and affecting OS. Additionally, the impact of subsequent therapies after disease progression also plays a crucial role in influencing OS. Patients who experience disease progression following first-line treatment with PD-1/PD-L1 inhibitors and TKIs may undergo second-line therapies that affect their OS outcomes. Variability in post-progression treatments among the included studies could have contributed to the observed lack of OS improvement. Moreover, the time needed for PD-1/PD-L1 inhibitors to generate a significant anti-tumor response may exceed the follow-up durations of some included studies. Extended follow-up periods might be required to fully capture the OS benefits.

The progression of cancer is intricately linked to its ability to circumvent immune surveillance. Checkpoint proteins play a crucial role in modulating the immune system’s response to both pathogens and tumor cells. Specifically, PD-1 impedes T-cell receptor signaling, curbing T-cell proliferation and the release of cytotoxic substances; sustained activation of PD-1 results in T-cell fatigue (Sen et al., 2016). Agents such as atezolizumab, camrelizumab, pembrolizumab, nivolumab, durvalumab, and tislelizumab, which inhibit PD-1 and PD-L1, have been shown to elicit objective tumor responses in approximately 15% of patients in phase 2 and 3 prospective trials (Sangro et al., 2021). The immunologic implications of TKIs have begun to be explored and remain incompletely elucidated. TKIs commonly target receptors for vascular endothelial growth factor (VEGF) and platelet-derived growth factor (PDGF), which are pivotal in their anti-angiogenic effects (Sampat and O'Neil, 2013). The inhibition of VEGF might also provoke immune-stimulating responses. TKIs can alter the immunological landscape of tumors, turning “cold” tumors “hot” and thereby broadening the cohort of patients who respond to checkpoint inhibitors due to unique immunomodulatory effects (Llovet et al., 2022). Experimental research has highlighted such transformations in the tumor microenvironment with the combination of pembrolizumab and lenvatinib in HCC, notably increasing the CD8 T-cell count while reducing regulatory T-cell numbers (Torrens et al., 2021). The combination of PD-1/PD-L1 inhibitors and multi-targeted TKIs is a VEGF-based method to enhance therapeutic efficacy. Beyond targeting the VEGF receptor, TKIs also interact with various other kinases, potentially influencing the effectiveness of PD-1/PD-L1 inhibitors (Rimassa et al., 2023). This synergistic interaction likely underpins the observed improvements in PFS and ORR with the combination therapy in our study. While no enhancements in OS or DCR were noted, more RCTs are necessary to further validate these findings.

Notably, our subgroup analysis revealed that combination therapy substantially enhanced OS in patients aged 65 years or older, of Asian descent, with an ECOG performance status of 1, BCLC stage C, baseline alpha-fetoprotein levels exceeding 400 ng/mL, and presenting with extrahepatic metastasis, macrovascular invasion, or HBV infection. Similarly, this therapeutic approach notably improved PFS in patients of male, Asian descent, with an ECOG performance status of 0, BCLC stage C, infected with HBV or hepatitis C virus (HCV), or exhibiting macrovascular invasion. These findings indicate that tailoring combination therapy to these specific demographics may enhance clinical outcomes. It is understood that chronic HBV infection leads to virus-specific T cell exhaustion, with the PD-1/PD-L1 pathway playing a critical role in inhibiting the activity of HBV-specific CD8^+^ T cells (Ye et al., 2015). Blocking PD-1/PD-L1 can, therefore, rejuvenate HBV-specific T-cell responses to viral antigens, potentially increasing the effectiveness of ICIs (Raziorrouh et al., 2010; Zhao et al., 2022). Conversely, non-viral HCC, including cases with hepatic steatosis, appears less responsive to immunotherapy compared to other HCC etiologies (Pfister et al., 2021). This pattern of response has been corroborated by studies like CheckMate 459 (Yau et al., 2022), KEYNOTE-240 (Finn et al., 2020c) and IMbrave150 (Finn et al., 2020b), where immunotherapy appeared less effective in patients with non-viral causes of HCC (Pfister et al., 2021). Furthermore, in subgroups with alpha-fetoprotein levels at or above 400 ng/mL, combination therapy also demonstrated a preference over the subgroups with alpha-fetoprotein less than 400 ng/mL in terms of both PFS and OS. The angiogenic nature of HCC and the association between high alpha-fetoprotein levels, increased VEGF expression, and immunosuppression might explain these outcomes (Galle et al., 2019). However, the scarcity of studies addressing these specific subgroup factors necessitates further investigation to elucidate the impact of immune-combination therapy on HCC treatment. Additionally, we established two subgroups based on the type of TKIs used in control treatments. Compared with first-line sorafenib monotherapy, the combination therapy of PD-1/PD-L1 inhibitors and TKIs significantly improved PFS, ORR, and DCR, but had no significant effect on OS. Similarly, compared to other TKI monotherapy (or plus placebo), adding PD-1/PD-L1 inhibitors to TKI monotherapy markedly improved PFS and OS, and increased ORR, but did not significantly influence DCR. Given the limited number of studies within these comparisons, further research is needed to refine and validate these findings.

The superior effectiveness of PD-1/PD-L1 inhibitors combined with TKIs in HBV-infected HCC patients can be linked to the distinct immune microenvironment shaped by chronic HBV infection. Chronic HBV is known to upregulate PD-L1 expression within the tumor microenvironment (Raziorrouh et al., 2014), potentially increasing the susceptibility of these tumors to PD-1/PD-L1 blockade. Furthermore, antiviral treatment in HBV-positive individuals may complement immunotherapy by lowering viral loads and mitigating inflammation, thereby restoring immune activity (Zheng et al., 2022). TKIs, through their antiangiogenic properties, may further augment the impact of ICIs by remodeling tumor vasculature and facilitating immune cell infiltration (Xing et al., 2021). Additionally, the enhanced outcomes of combination therapy observed in Asian populations can be attributed to several factors. First, HBV infection, the leading cause of HCC in Asian patients, is associated with elevated PD-L1 levels and a more immunogenic tumor milieu (Xuan Hoan et al., 2022). Second, genetic and pharmacokinetic variations in this population, including differences in drug-metabolizing enzymes and immune-related gene polymorphisms, may boost responsiveness to PD-1/PD-L1 inhibitors and TKIs. Moreover, the prevalent use of antiviral therapies and early detection strategies in Asian regions likely contributes to more favorable responses to combination treatments. These findings highlight the importance of understanding the underlying mechanisms driving enhanced efficacy in HBV-infected and Asian patients. A deeper understanding of these biological and clinical factors could inform patient stratification and optimize treatment strategies for advanced HCC. Future studies should explore the genetic, immunological, and pharmacological factors that contribute to these observed differences, with the goal of developing personalized treatment approaches.

Besides therapeutic efficacy, TRAEs warrant close scrutiny (Zeng et al., 2023). In our research, a majority of participants from both the experimental and control arms reported experiencing TRAEs. The use of PD-1/PD-L1 inhibitors combined with TKIs led to a higher incidence of serious and grade ≥3 TRAEs compared to the control regimen. Across the included 4 studies, prevalent TRAEs observed in combination and control therapies included hypertension, elevated AST, proteinuria, increased ALT, reduced platelet counts, increased blood bilirubin, palmar-plantar erythrodysaesthesia syndrome, diarrhea, fatigue, rash, reduced appetite, weight decreased, asthenia, nausea, and increased lipase levels. Notably, the combination therapy group showed a significant uptick in cases of elevated AST, ALT, and blood bilirubin compared to controls. Although the majority of TRAEs were classified as grade 1–2, suggesting manageability, the elevated risk of AEs highlights the imperative for rigorous monitoring and proactive management of these toxicities. The engagement of multidisciplinary care teams, encompassing hepatologists, oncologists, and supportive care professionals, is vital for enhancing patient outcomes and sustaining quality of life (QoL) throughout the treatment process (Xu and Sun, 2022).

This study has several limitations. First, this meta-analysis did not incorporate individual patient data, leading to an inherent selection bias. Second, our analysis only encompassed 4 studies that compared the efficacy and safety of PD-1/PD-L1 inhibitors combined with TKIs against first-line monotherapy or TKI monotherapy in patients with advanced or unresectable HCC. More comprehensive clinical trials are needed to generate robust data that could be included in subsequent analyses. Third, the RCTs included in this meta-analysis featured a variety of therapeutic agents and had diverse patient baseline characteristics, such as age, sex, region, race, ECOG performance status, BCLC stage, baseline alpha-fetoprotein levels, disease etiology, macrovascular invasion, Child-Pugh classification, and extrahepatic metastasis. These factors could potentially introduce significant heterogeneity in the analysis of clinical outcomes and TRAEs. Thus, subgroup analyses were performed to categorize data based on baseline characteristics, aiming to reduce the effects of heterogeneity. Future research could more comprehensively explore the efficacy and safety of the combination therapy through further subgroup analyses, such as PD-L1 expression levels and Albumin-Bilirubin (ALBI) grade, or by employing network meta-analysis. Fourth, the RCTs analyzed in this study did not report QoL outcomes, despite QoL being a critical factor in the management of advanced HCC. The lack of QoL information hinders a comprehensive evaluation of the benefit-risk profile of combination therapies when compared to TKI monotherapy or other established first-line options. Future RCTs should prioritize the collection and reporting of QoL outcomes using standardized and validated instruments to provide a more holistic evaluation of treatment efficacy and safety.

## 5 Conclusion

In conclusion, the combination of PD-1/PD-L1 inhibitors with TKIs emerges as a promising therapeutic option for advanced or unresectable HCC. This meta-analysis has demonstrated the efficacy of this combination therapy in enhancing PFS and ORR, and for the first time, identified better survival benefits among patients with HBV infection and within the Asian demographic. Nonetheless, the associated increase in serious and grade ≥3 TRAEs demands rigorous patient selection and management strategies. Future studies should concentrate on optimizing treatment protocols and investigate new therapeutic combinations.

## Data Availability

The original contributions presented in the study are included in the article/Supplementary Material. Further inquiries can be directed to the corresponding author.
