# Supplementary material for: Efficacy and safety of PD-1/PD-L1 inhibitors combined with tyrosine kinase inhibitors as first-line treatment for hepatocellular carcinoma: a meta-analysis and trial sequential analysis of randomized controlled trials
Source: Front Pharmacol. 2025 Mar 24;16:1535444. doi: 10.3389/fphar.2025.1535444 (PMC11973308; doi:10.3389/fphar.2025.1535444)
Supplement: Supplementary file 1 [file Supplementaryfile1.docx]

| **PubMed 1535** |
| --- |
| #1 (PD-1 inhibitors) OR (PD-L1 inhibitors) OR (immune checkpoint inhibitors) OR (pembrolizumab) OR (atezolizumab) OR (nivolumab) OR (durvalumab) OR (camrelizumab) OR (tislelizumab) OR (avelumab) OR (toripalimab) OR (sintilimab) OR (penpulimab) OR (zimberelimab) OR (serplulimab) OR (sugemalimab) OR (pucotenlimab) OR (envafolimab) |
| #2 (tyrosine kinase inhibitors) OR (TKIs) OR (sorafenib) OR (lenvatinib) OR (sunitinib) OR (cabozantinib) OR (rivoceranib) OR (apatinib) OR (regorafenib) OR (donafenib) OR (linifanib) |
| #3 (hepatocellular carcinoma) OR (liver cancer) OR (liver neoplasms) OR (hepatocarcinoma) OR (HCC) OR (liver cell carcinoma) |
| #4 #1 AND #2 AND #3 |
| **Web of Science 2506** |
| #1 TS=((PD-1 inhibitors) OR (PD-L1 inhibitors) OR (immune checkpoint inhibitors) OR (pembrolizumab) OR (atezolizumab) OR (nivolumab) OR (durvalumab) OR (camrelizumab) OR (tislelizumab) OR (avelumab) OR (toripalimab) OR (sintilimab) OR (penpulimab) OR (zimberelimab) OR (serplulimab) OR (sugemalimab) OR (pucotenlimab) OR (envafolimab)) |
| #2 TS=((tyrosine kinase inhibitors) OR (TKIs) OR (sorafenib) OR (lenvatinib) OR (sunitinib) OR (cabozantinib) OR (rivoceranib) OR (apatinib) OR (regorafenib) OR (donafenib) OR (linifanib)) |
| #3 TS=((hepatocellular carcinoma) OR (liver cancer) OR (liver neoplasms) OR (hepatocarcinoma) OR (HCC) OR (liver cell carcinoma)) |
| #4 #1 AND #2 AND #3 |
| **The Cochrane Library 484** |
| #1 All Text=((PD-1 inhibitors) OR (PD-L1 inhibitors) OR (immune checkpoint inhibitors) OR (pembrolizumab) OR (atezolizumab) OR (nivolumab) OR (durvalumab) OR (camrelizumab) OR (tislelizumab) OR (avelumab) OR (toripalimab) OR (sintilimab) OR (penpulimab) OR (zimberelimab) OR (serplulimab) OR (sugemalimab) OR (pucotenlimab) OR (envafolimab)) |
| #2 All Text=((tyrosine kinase inhibitors) OR (TKIs) OR (sorafenib) OR (lenvatinib) OR (sunitinib) OR (cabozantinib) OR (rivoceranib) OR (apatinib) OR (regorafenib) OR (donafenib) OR (linifanib)) |
| #3 All Text=((hepatocellular carcinoma) OR (liver cancer) OR (liver neoplasms) OR (hepatocarcinoma) OR (HCC) OR (liver cell carcinoma)) |
| #4 #1 AND #2 AND #3 |
| **Embase 2249** |
| #1 'pd-1 inhibitors':ti,ab,kw OR 'pd-l1 inhibitors':ti,ab,kw OR 'immune checkpoint inhibitors':ti,ab,kw OR pembrolizumab:ti,ab,kw OR atezolizumab:ti,ab,kw OR nivolumab:ti,ab,kw OR durvalumab:ti,ab,kw OR camrelizumab:ti,ab,kw OR tislelizumab:ti,ab,kw OR avelumab:ti,ab,kw OR toripalimab:ti,ab,kw OR sintilimab:ti,ab,kw OR penpulimab:ti,ab,kw OR zimberelimab:ti,ab,kw OR serplulimab:ti,ab,kw OR sugemalimab:ti,ab,kw OR pucotenlimab:ti,ab,kw OR envafolimab:ti,ab,kw |
| #2 'tyrosine kinase inhibitors':ti,ab,kw OR tkis:ti,ab,kw OR sorafenib:ti,ab,kw OR lenvatinib:ti,ab,kw OR sunitinib:ti,ab,kw OR cabozantinib:ti,ab,kw OR rivoceranib:ti,ab,kw OR apatinib:ti,ab,kw OR regorafenib:ti,ab,kw OR donafenib:ti,ab,kw OR linifanib:ti,ab,kw |
| #3 'hepatocellular carcinoma':ti,ab,kw OR 'liver cancer':ti,ab,kw OR 'liver neoplasms':ti,ab,kw OR hepatocarcinoma:ti,ab,kw OR hcc:ti,ab,kw OR 'liver cell carcinoma':ti,ab,kw |
| #4 #1 AND #2 AND #3 |
