# Supplementary material for: Efficacy and safety of PD-1/PD-L1 inhibitors combined with tyrosine kinase inhibitors as first-line treatment for hepatocellular carcinoma: a meta-analysis and trial sequential analysis of randomized controlled trials
Source: Front Pharmacol. 2025 Mar 24;16:1535444. doi: 10.3389/fphar.2025.1535444 (PMC11973308; doi:10.3389/fphar.2025.1535444)
Supplement: Supplementary file 2 [file Table1.docx]

| TABLE S1 Quality analysis of the included RCTs by modified Jadad scale. | | | | | | |
| --- | --- | --- | --- | --- | --- | --- |
| Study | Randomization | Randomization concealment | Double blind | Withdrawals and dropouts | Score | Study quality |
| Qin (2023) | 2 | 2 | 0 | 1 | 5 | High |
| Llovet (2023) | 2 | 2 | 2 | 1 | 7 | High |
| Yau (2024) | 2 | 2 | 0 | 1 | 5 | High |
| Kelley (2022) | 2 | 2 | 0 | 1 | 5 | High |
