# Supplementary material for: Efficacy and safety of PD-1/PD-L1 inhibitors combined with tyrosine kinase inhibitors as first-line treatment for hepatocellular carcinoma: a meta-analysis and trial sequential analysis of randomized controlled trials
Source: Front Pharmacol. 2025 Mar 24;16:1535444. doi: 10.3389/fphar.2025.1535444 (PMC11973308; doi:10.3389/fphar.2025.1535444)
Supplement: Supplementary file 3 [file Image1.pdf]

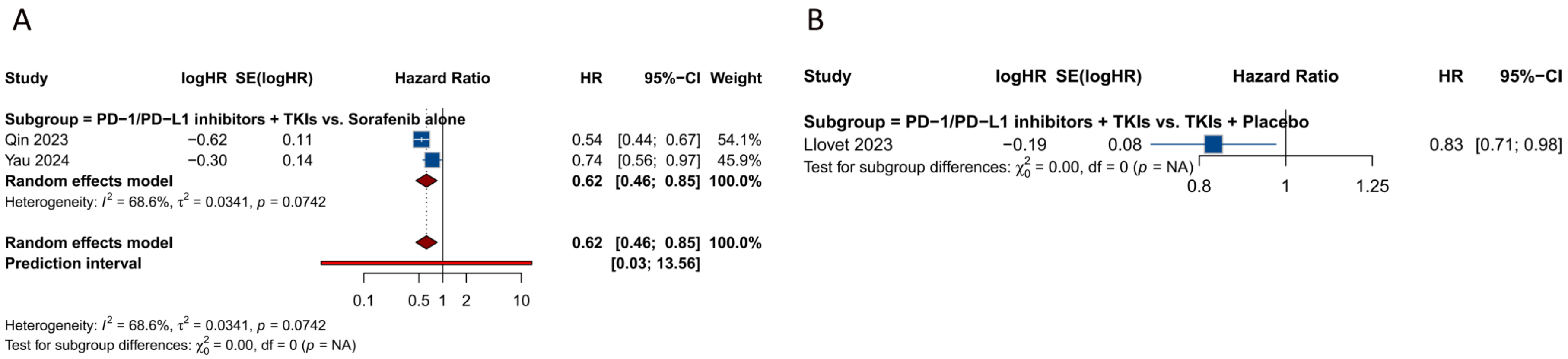

FIGURE S1 Subgroup analysis of progression-free survival based on the types of tyrosine kinase inhibitors used in the control group. (A) PD-1/PD-L1 inhibitors + TKIs vs. Sorafenib alone; (B) PD-1/PD-L1 inhibitors + TKIs vs. TKIs + Placebo.

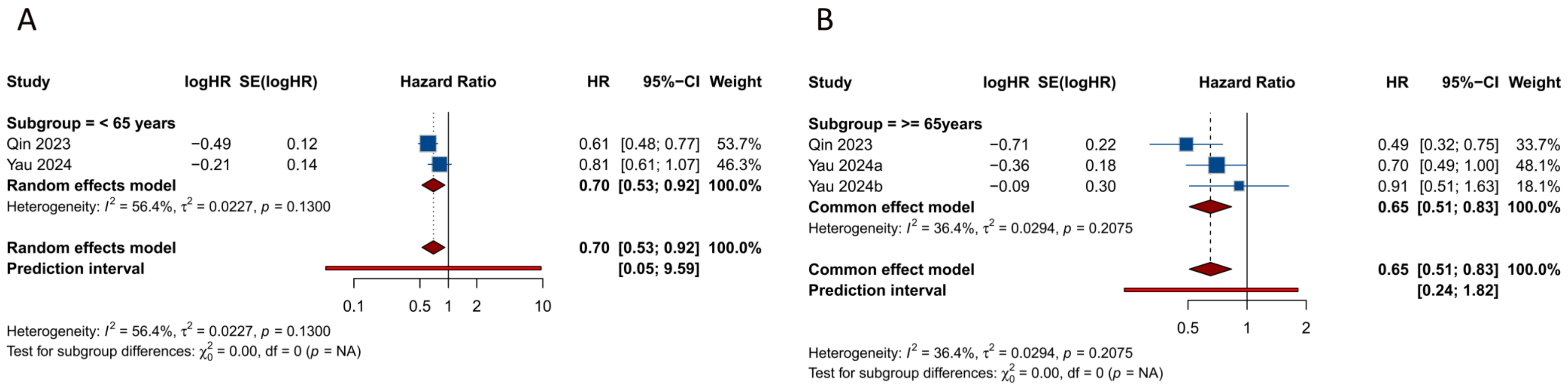

FIGURE S2 Subgroup analysis of progression-free survival based the age of participants. (A) < 65 years; (B) ≥ 65 years.

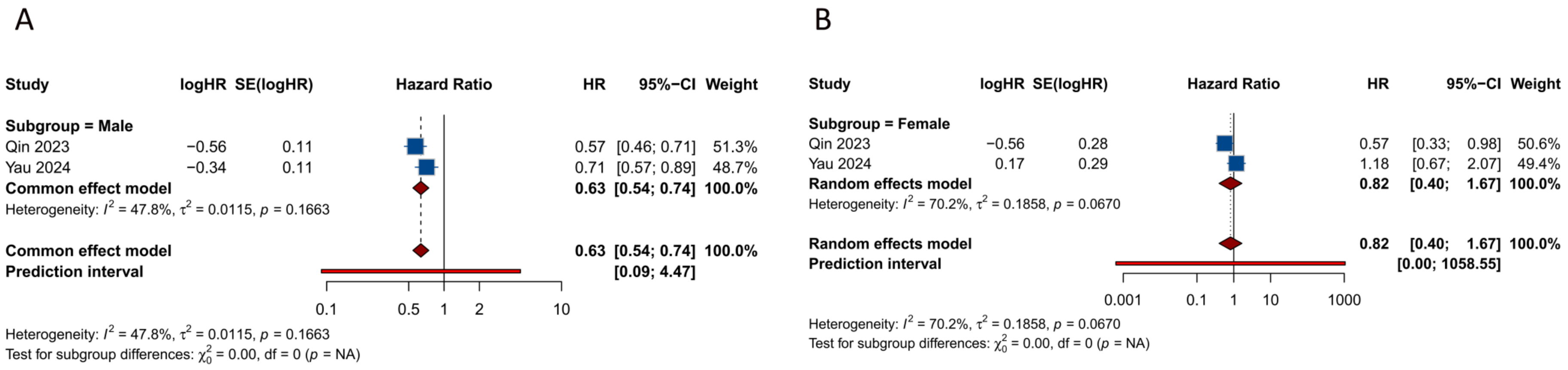

FIGURE S3 Subgroup analysis of progression-free survival based on the gender of participants. (A) Male; (B) Female.

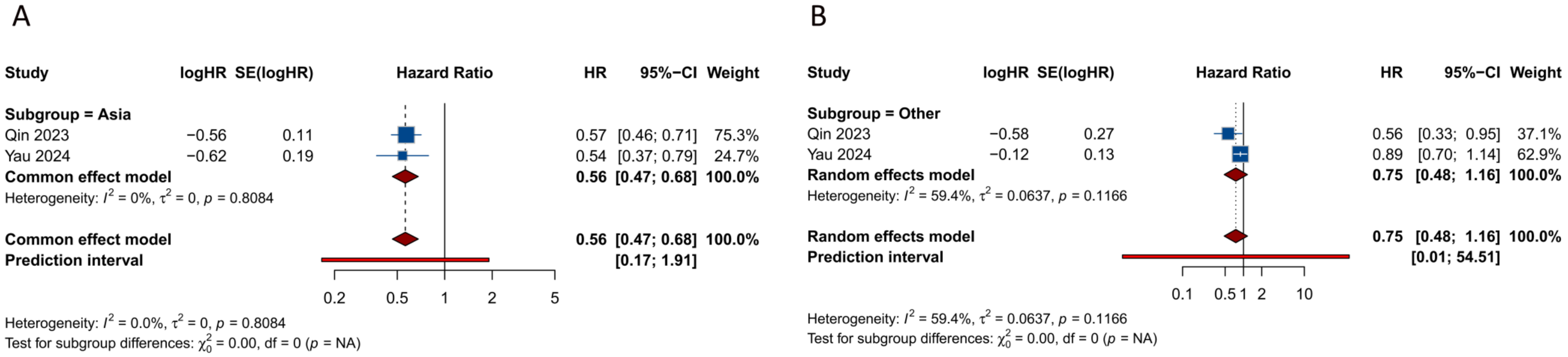

FIGURE S4 Subgroup analysis of progression-free survival based on region. (A) Asia; (B) Other.

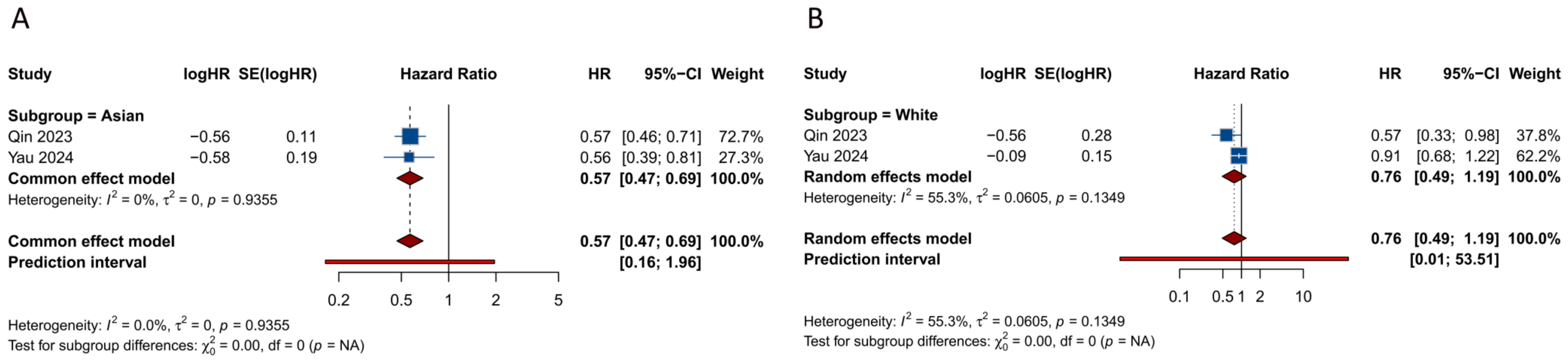

FIGURE S5 Subgroup analysis of progression-free survival based on the race of participants. (A) Asian; (B) White.

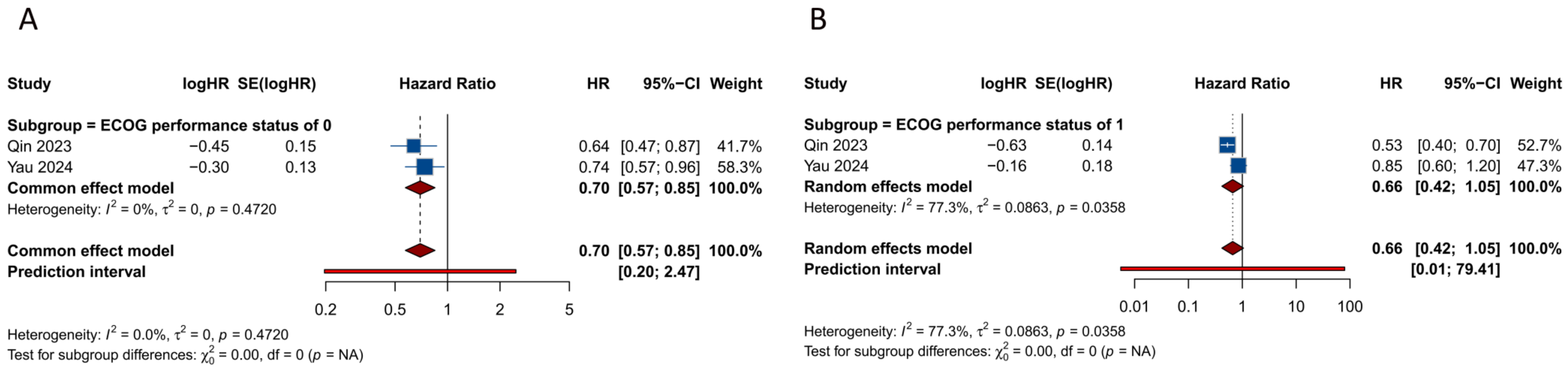

FIGURE S6 Subgroup analysis of progression-free survival based on Eastern Cooperative Oncology Group (ECOG) performance status (PS). (A) ECOG PS of 0; (B) ECOG PS of 1.

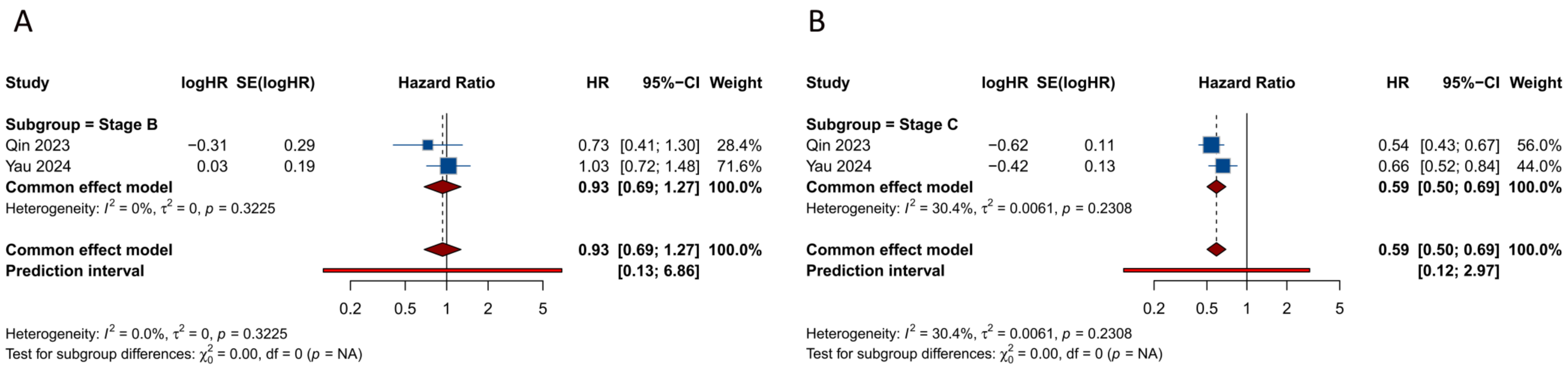

FIGURE S7 Subgroup analysis of progression-free survival based on Barcelona Clinic Liver Cancer (BCLC) stage. (A) Stage B; (B) Stage C.

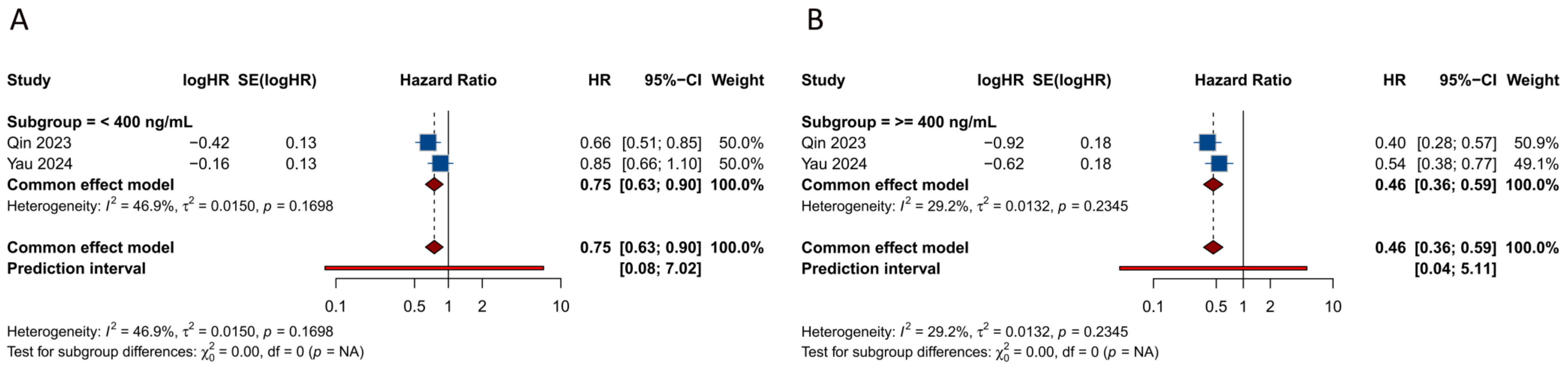

FIGURE S8 Subgroup analysis of progression-free survival based on baseline alpha-fetoprotein (ng/mL). (A) < 400; (B) ≥ 400.

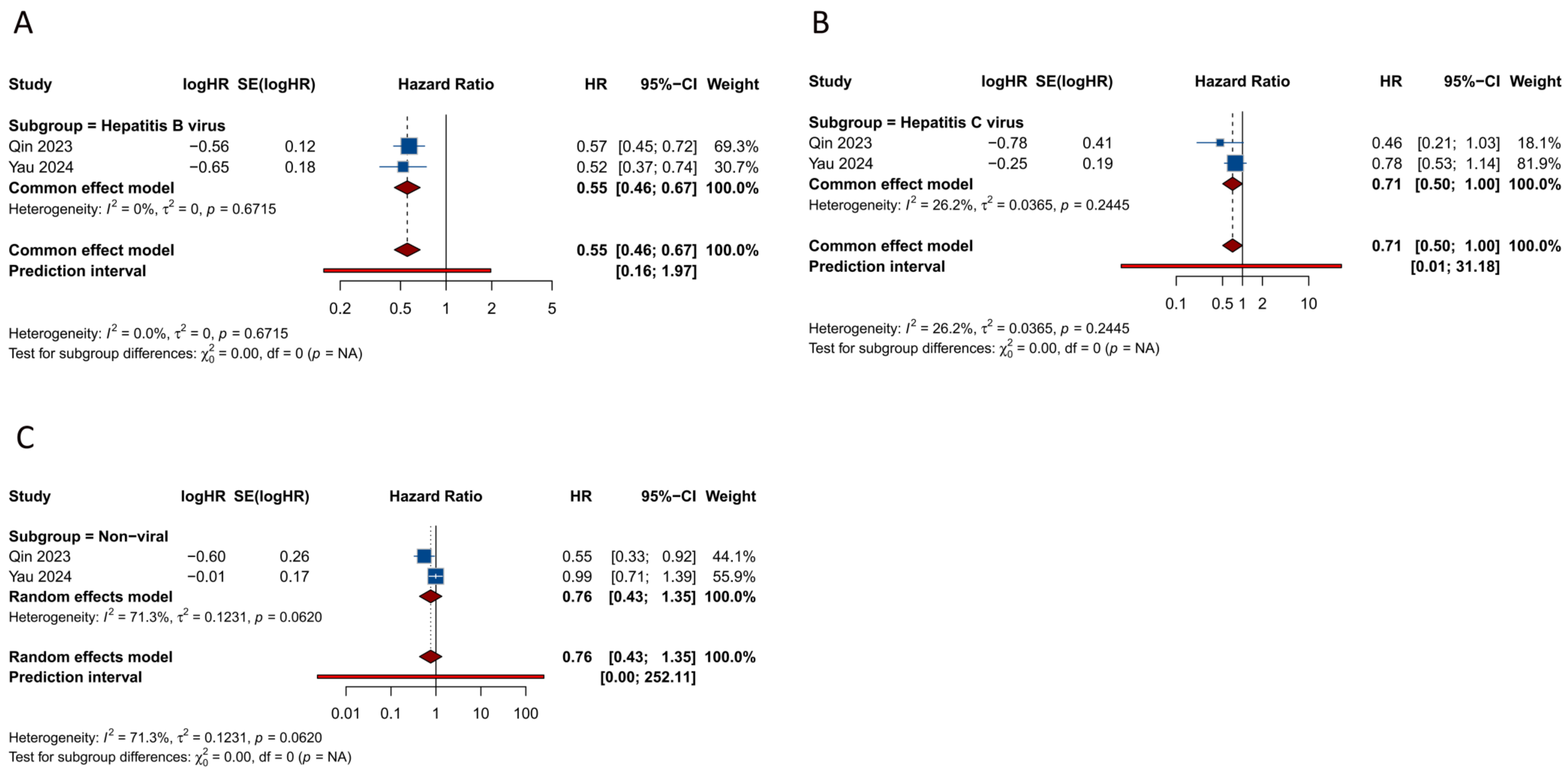

FIGURE S9 Subgroup analysis of progression-free survival based on disease aetiology. (A) Hepatitis B virus; (B) Hepatitis C virus; (C) Non-viral.

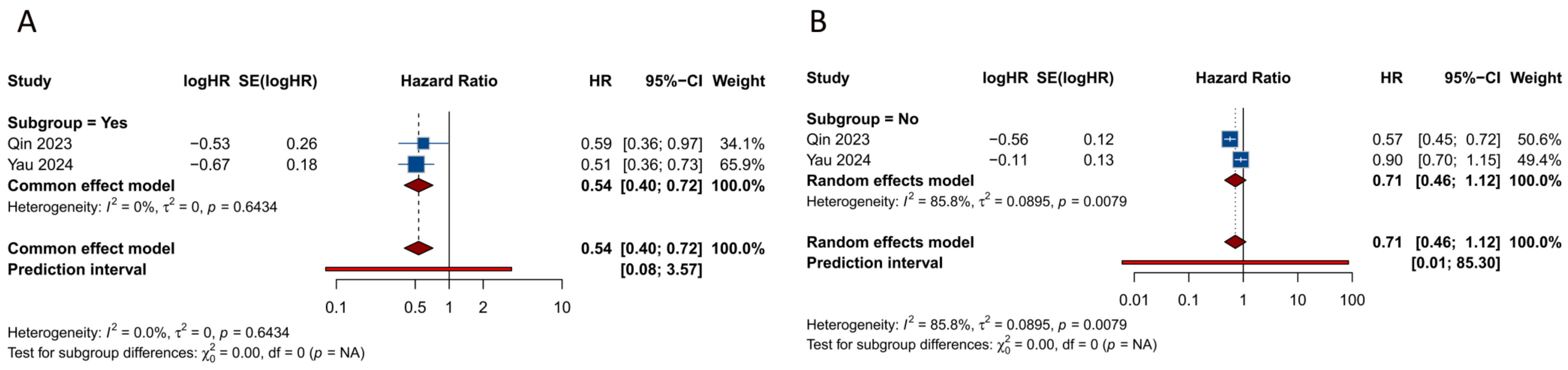

FIGURE S10 Subgroup analysis of progression-free survival based on macrovascular invasion. (A) Macrovascular invasion = Yes; (B) Macrovascular invasion = No.

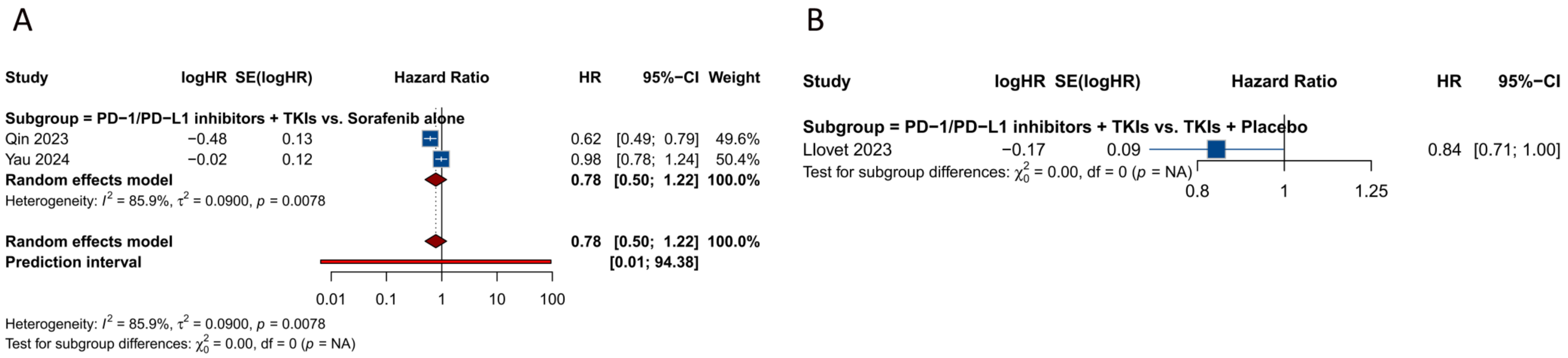

FIGURE S11 Subgroup analysis of overall survival based on the types of tyrosine kinase inhibitors used in the control group. (A) PD-1/PD-L1 inhibitors + TKIs vs. Sorafenib alone; (B) PD-1/PD-L1 inhibitors + TKIs vs. TKIs + Placebo.

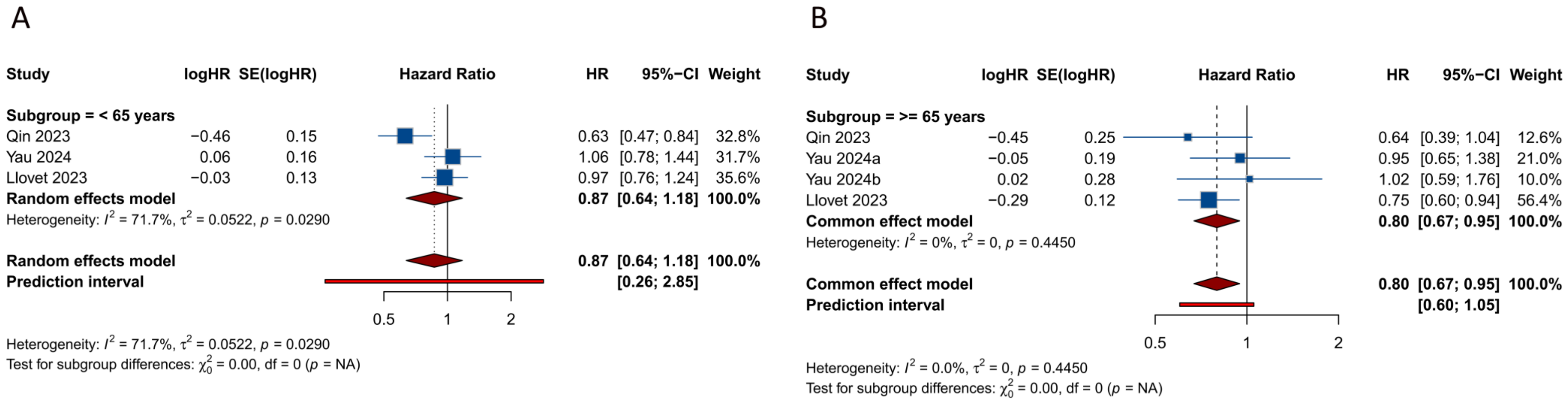

FIGURE S12 Subgroup analysis of overall survival based on the age of participants. (A) < 65 years; (B) ≥ 65 years.

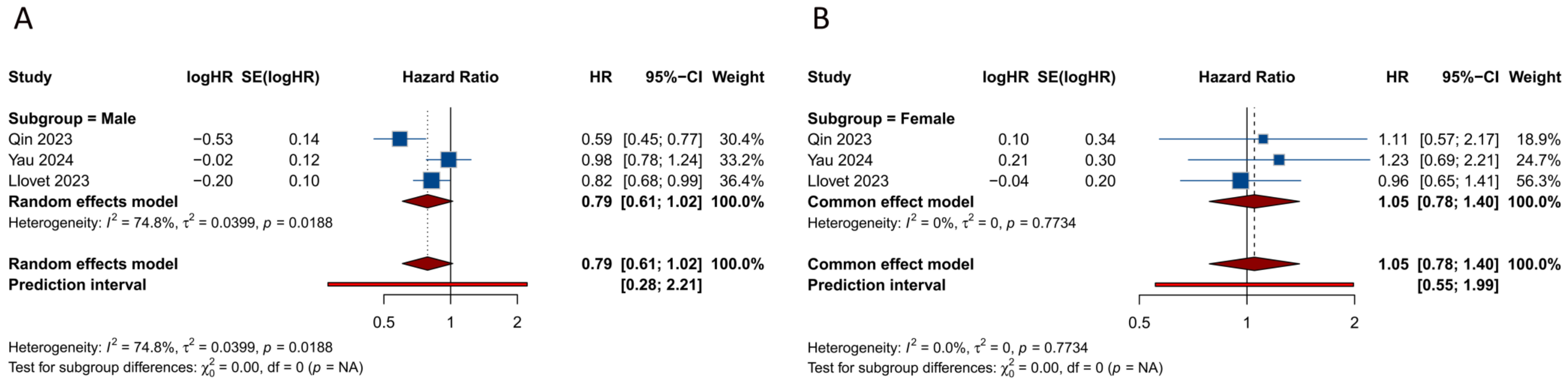

FIGURE S13 Subgroup analysis of overall survival based on the gender of participants. (A) Male; (B) Female.

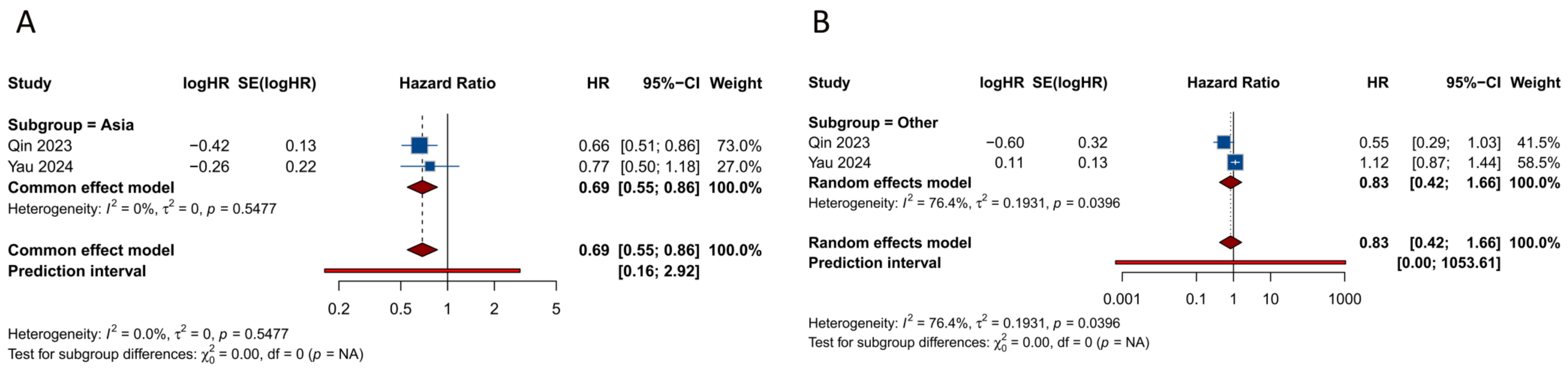

FIGURE S14 Subgroup analysis of overall survival based on region. (A) Asia; (B) Other.

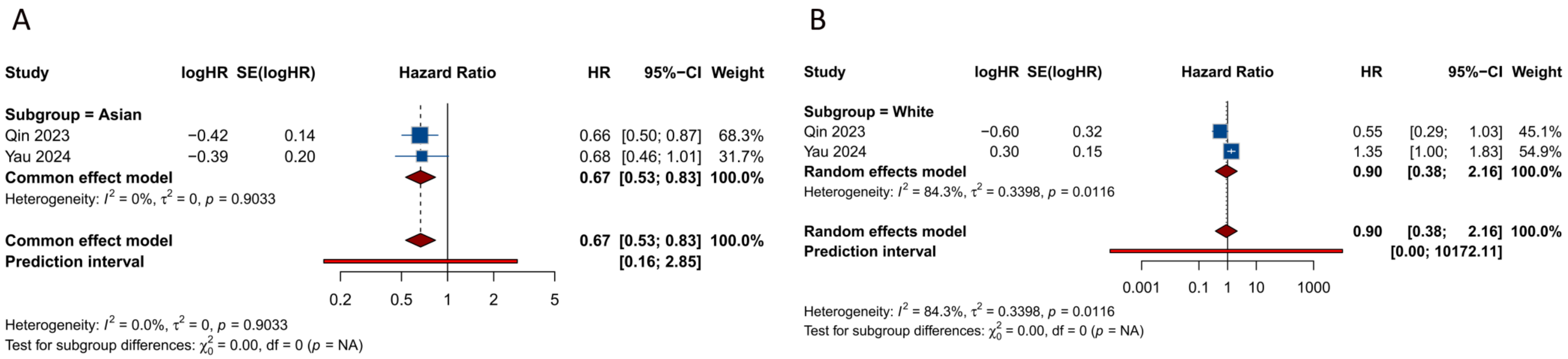

FIGURE S15 Subgroup analysis of overall survival based on the race of participants. (A) Asian; (B) White.

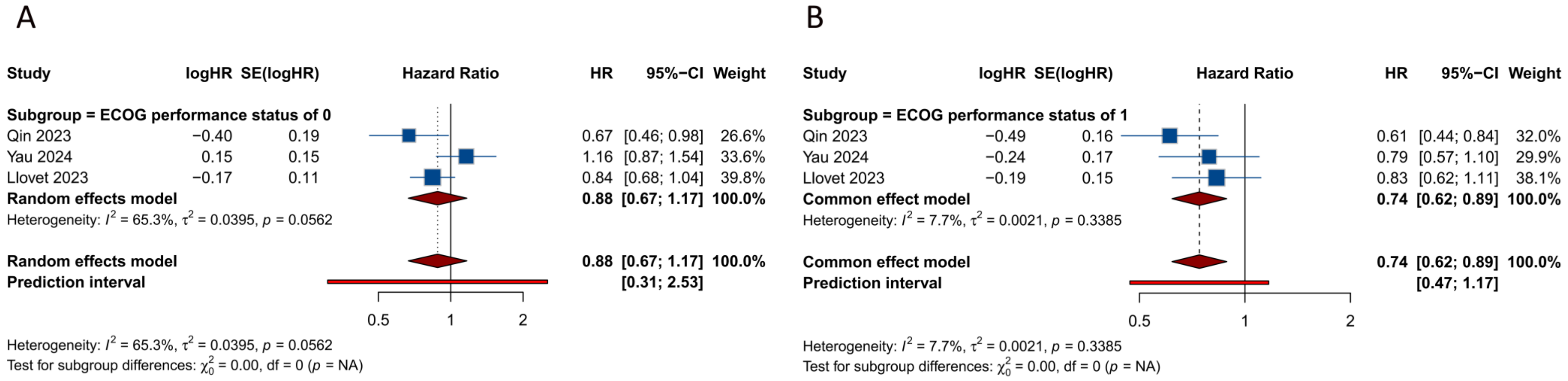

FIGURE S16 Subgroup analysis of overall survival based on Eastern Cooperative Oncology Group (ECOG) performance status (PS). (A) ECOG PS of 0; (B) ECOG PS of 1.

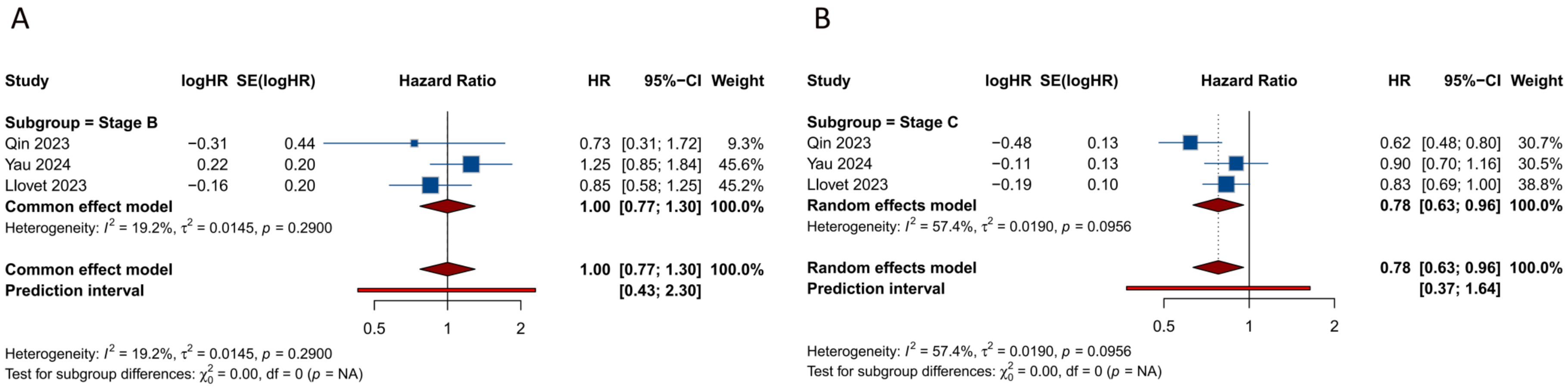

FIGURE S17 Subgroup analysis of overall survival based on Barcelona Clinic Liver Cancer (BCLC) stage. (A) Stage B; (B) Stage C.

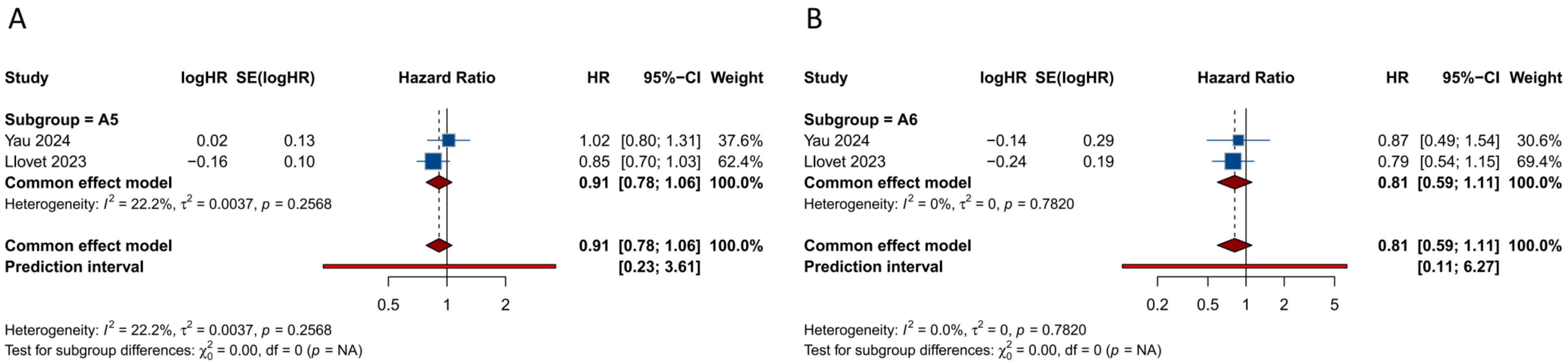

FIGURE S18 Subgroup analysis of overall survival based on Child-Pugh classification. (A) A5; (B) A6.

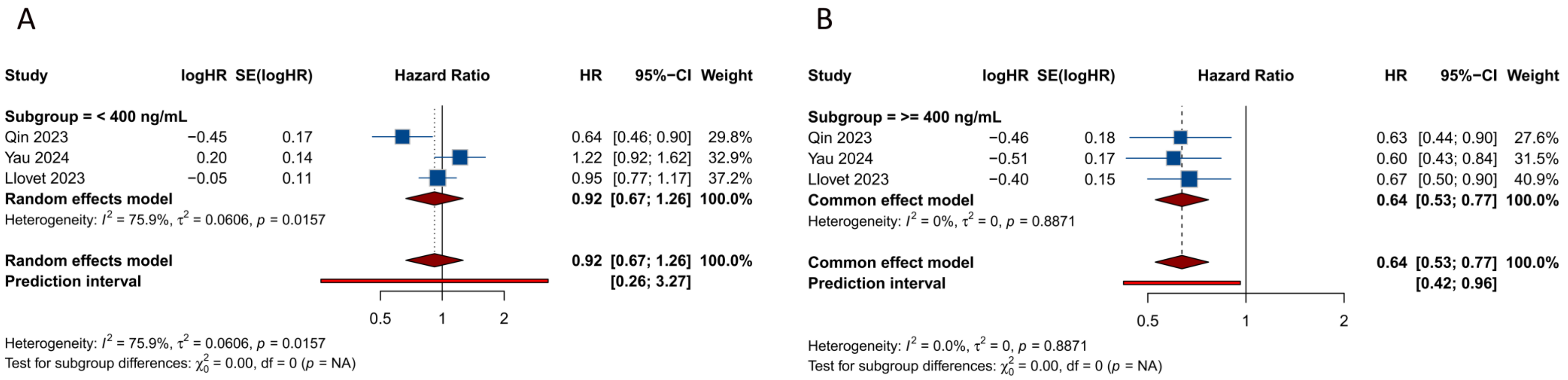

FIGURE S19 Subgroup analysis of overall survival based on baseline alpha-fetoprotein (ng/mL). (A) < 400; (B) ≥ 400.

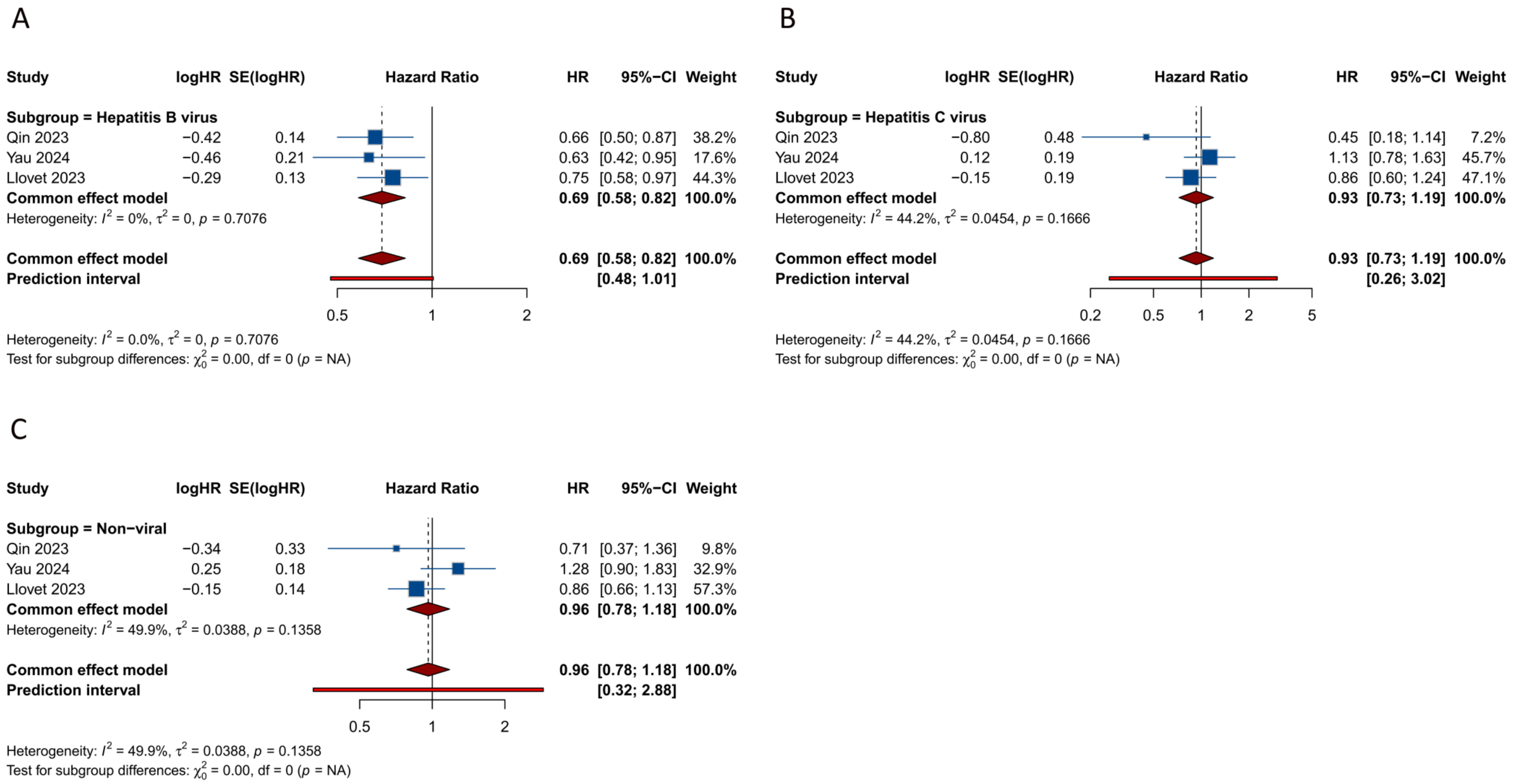

FIGURE S20 Subgroup analysis of overall survival based on disease aetiology. (A) Hepatitis B virus; (B) Hepatitis C virus; (C) Non-viral.

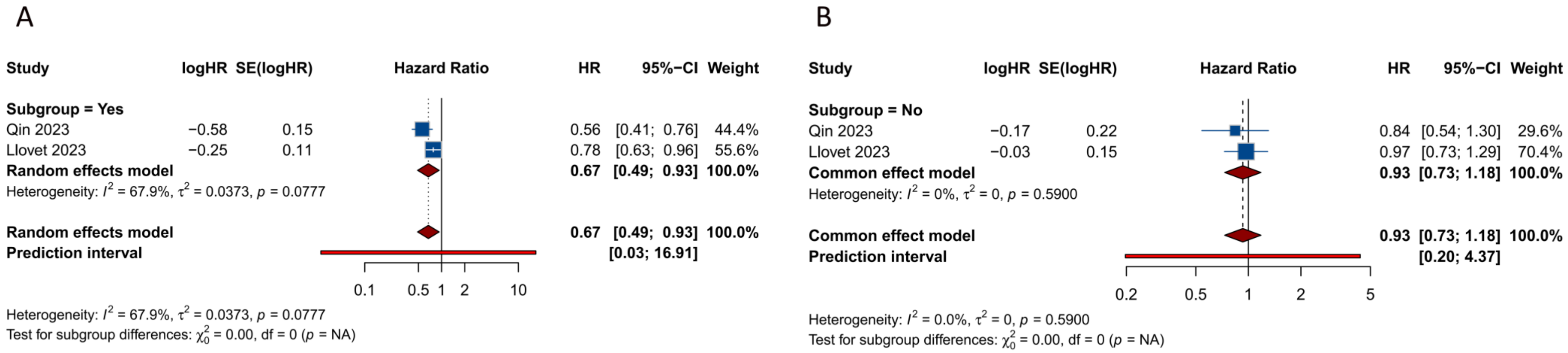

FIGURE S21 Subgroup analysis of overall survival based on extrahepatic metastasis. (A) Extrahepatic metastasis = Yes; (B) Extrahepatic metastasis = No.

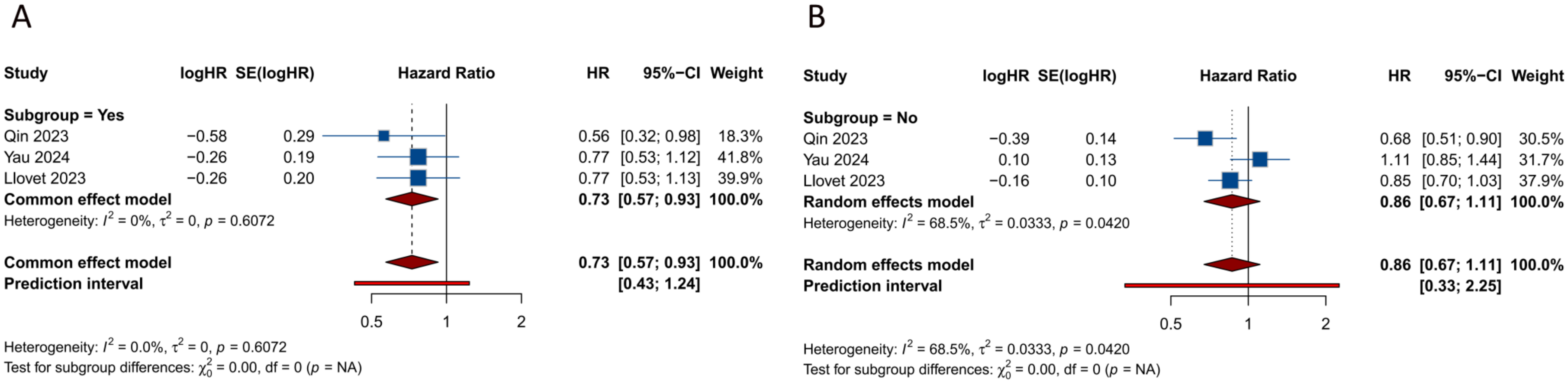

FIGURE S22 Subgroup analysis of overall survival based on macrovascular invasion. (A) Macrovascular invasion = Yes; (B) Macrovascular invasion = No.

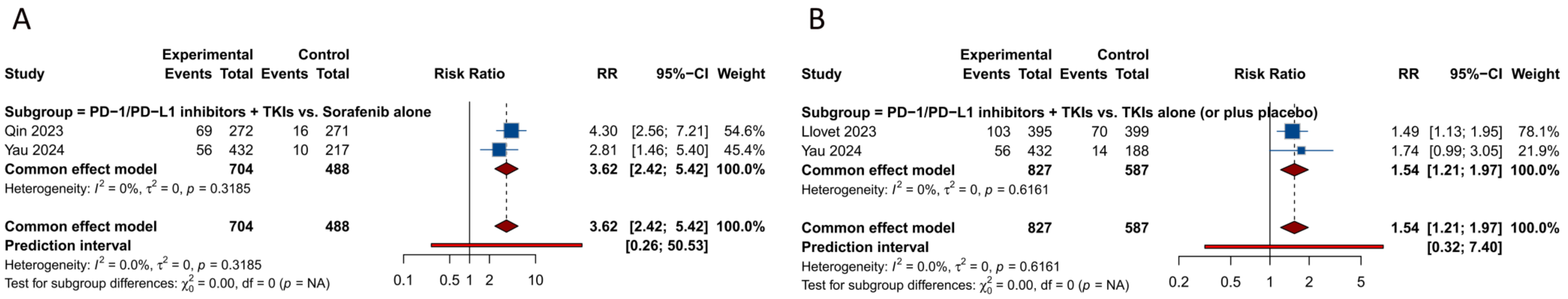

FIGURE S23 Subgroup analysis of objective response rate based on the types of tyrosine kinase inhibitors used in the control group. (A) PD-1/PD-L1 inhibitors + TKIs vs. Sorafenib alone; (B) PD-1/PD-L1 inhibitors + TKIs vs. TKIs alone (or plus placebo).

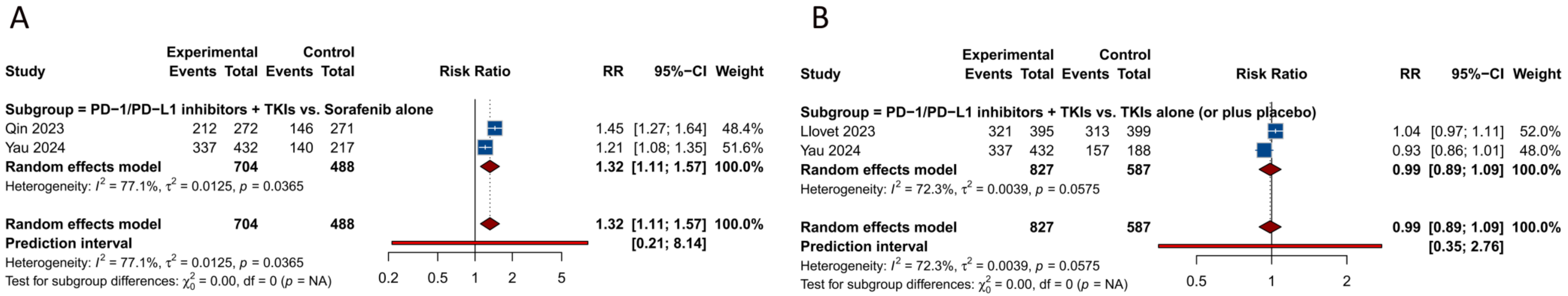

FIGURE S24 Subgroup analysis of disease control rate based on the types of tyrosine kinase inhibitors used in the control group. (A) PD-1/PD-L1 inhibitors + TKIs vs. Sorafenib alone; (B) PD-1/PD-L1 inhibitors + TKIs vs. TKIs alone (or plus placebo).

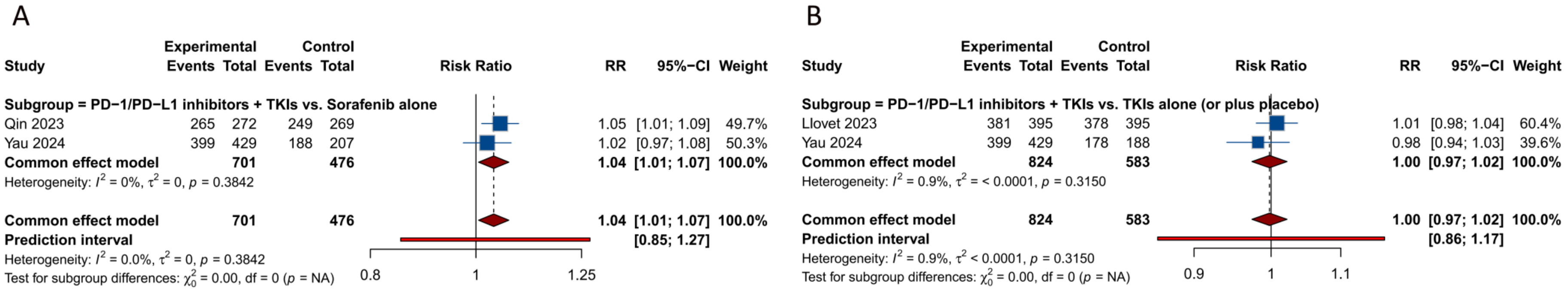

FIGURE S25 Subgroup analysis of any grade treatment-related adverse events based on the types of tyrosine kinase inhibitors used in the control group. (A) PD-1/PD-L1 inhibitors + TKIs vs. Sorafenib alone; (B) PD-1/PD-L1 inhibitors + TKIs vs. TKIs alone (or plus placebo).

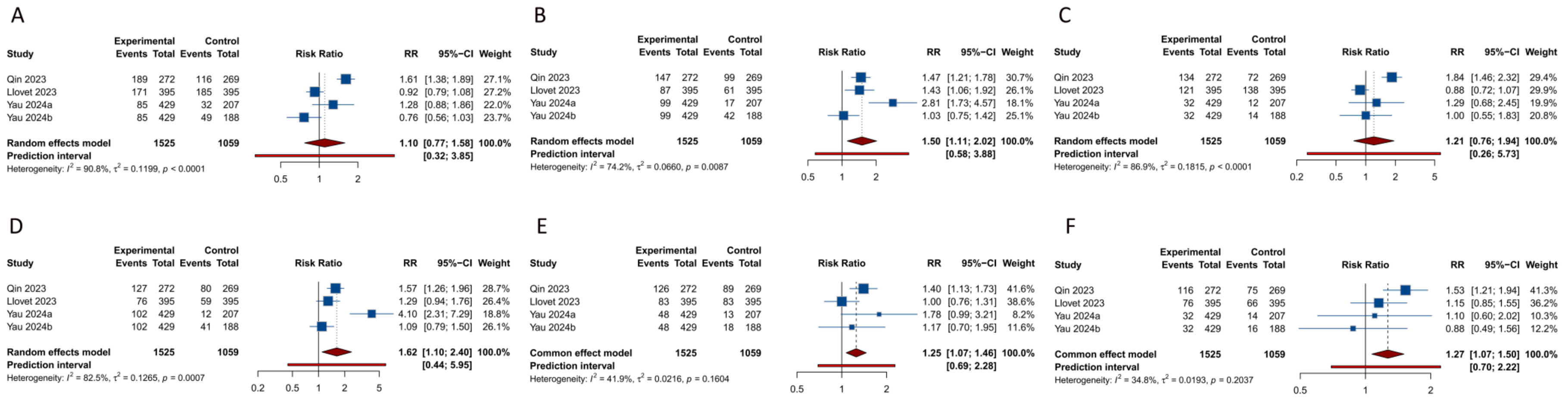

FIGURE S26 Forest plots of specific any grade treatment-related adverse events. (A) Hypertension; (B) Aspartate aminotransferase increased; (C) Proteinuria; (D) Alanine aminotransferase increased; (E) Platelet count decreased; (F) Blood bilirubin increased.

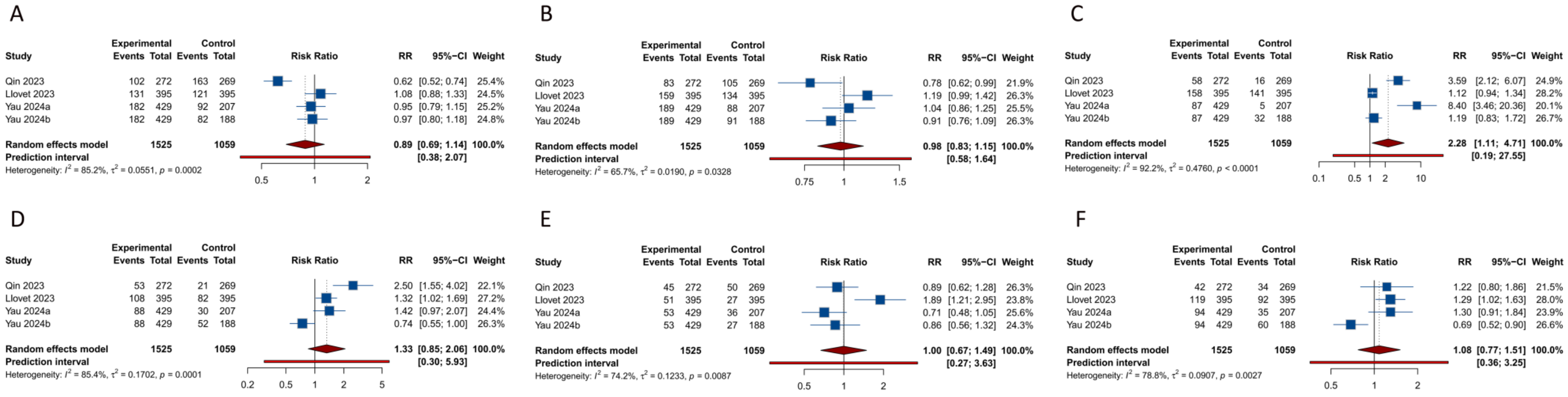

FIGURE S27 Forest plots of specific any grade treatment-related adverse events. (A) Palmar-plantar erythrodysaesthesia syndrome; (B) Diarrhoea; (C) Hypothyroidism; (D) Fatigue; (E) Rash; (F) Decreased appetite.

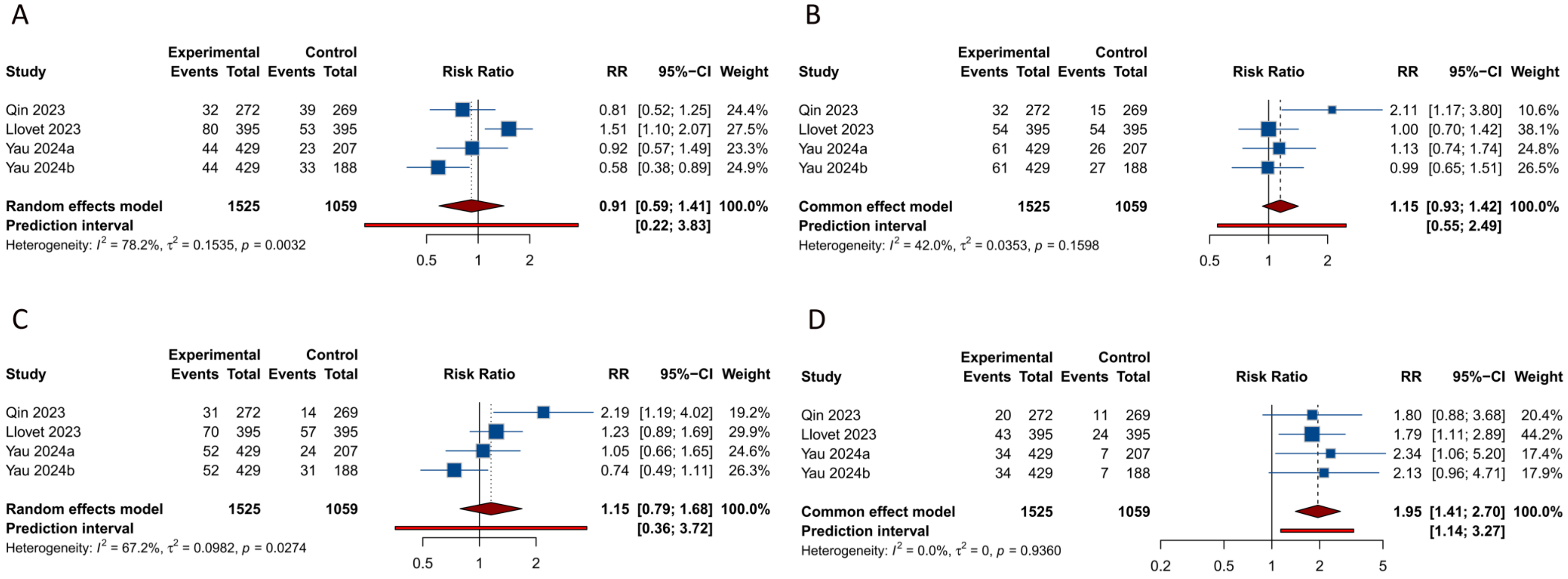

FIGURE S28 Forest plots of specific any grade treatment-related adverse events. (A) Weight decreased; (B) Asthenia; (C) Nausea; (D) Lipase increased.

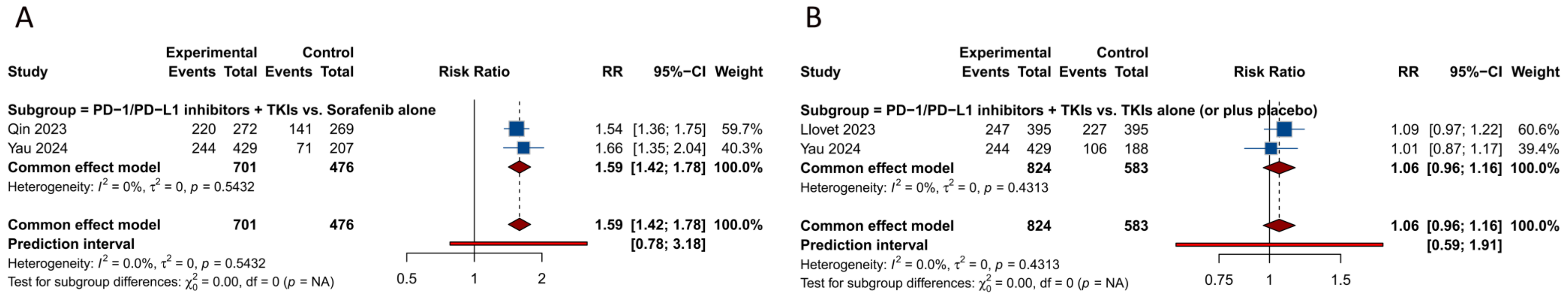

FIGURE S29 Subgroup analysis of grade ≥ 3 treatment-related adverse events based on the types of tyrosine kinase inhibitors used in the control group. (A) PD-1/PD-L1 inhibitors + TKIs vs. Sorafenib alone; (B) PD-1/PD-L1 inhibitors + TKIs vs. TKIs alone (or plus placebo).

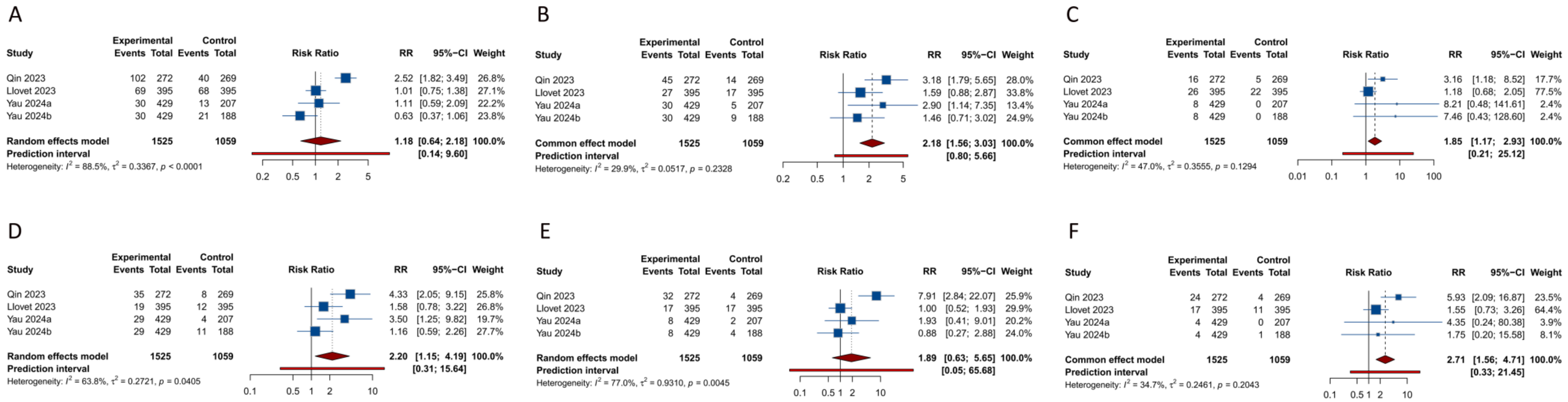

FIGURE S30 Forest plots of specific grade ≥ 3 treatment-related adverse events. (A) Hypertension; (B) Aspartate aminotransferase increased; (C) Proteinuria; (D) Alanine aminotransferase increased; (E) Platelet count decreased; (F) Blood bilirubin increased.

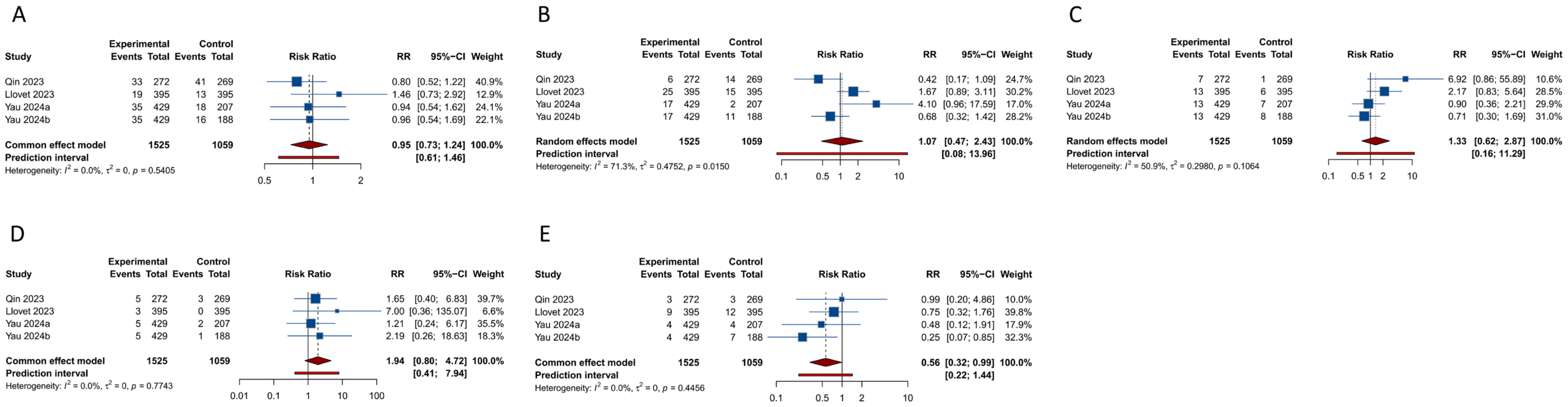

FIGURE S31 Forest plots of specific grade ≥ 3 treatment-related adverse events. (A) Palmar-plantar erythrodysaesthesia syndrome; (B) Diarrhoea; (C) Fatigue; (D) Rash; (E) Decreased appetite.

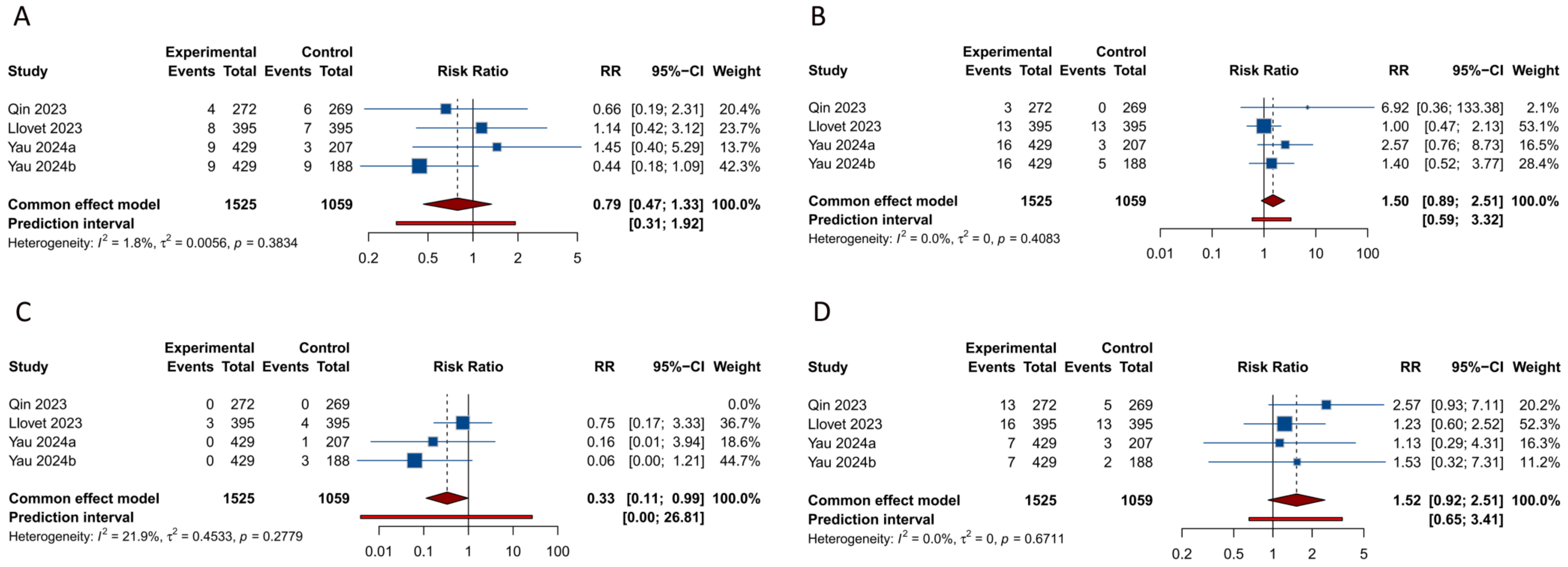

FIGURE S32 Forest plots of specific grade ≥ 3 treatment-related adverse events. (A) Weight decreased; (B) Asthenia; (C) Nausea; (D) Lipase increased.

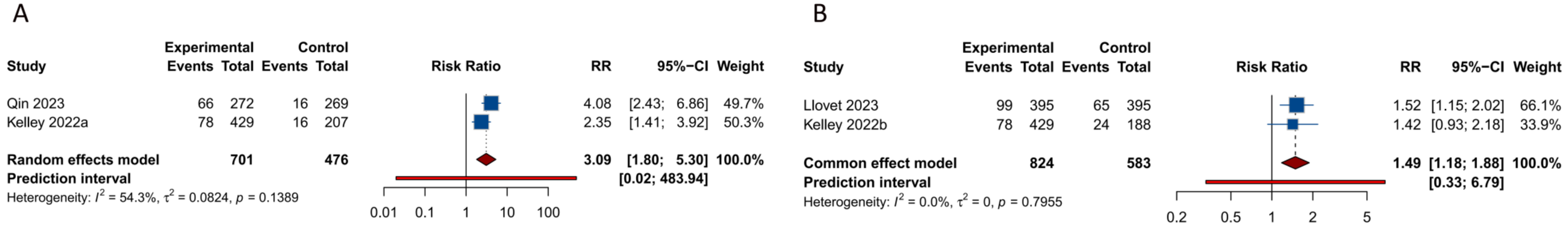

FIGURE S33 Subgroup analysis of serious treatment-related adverse events based on the types of tyrosine kinase inhibitors used in the control group. (A) PD-1/PD-L1 inhibitors + TKIs vs. Sorafenib alone; (B) PD-1/PD-L1 inhibitors + TKIs vs. TKIs alone (or plus placebo).

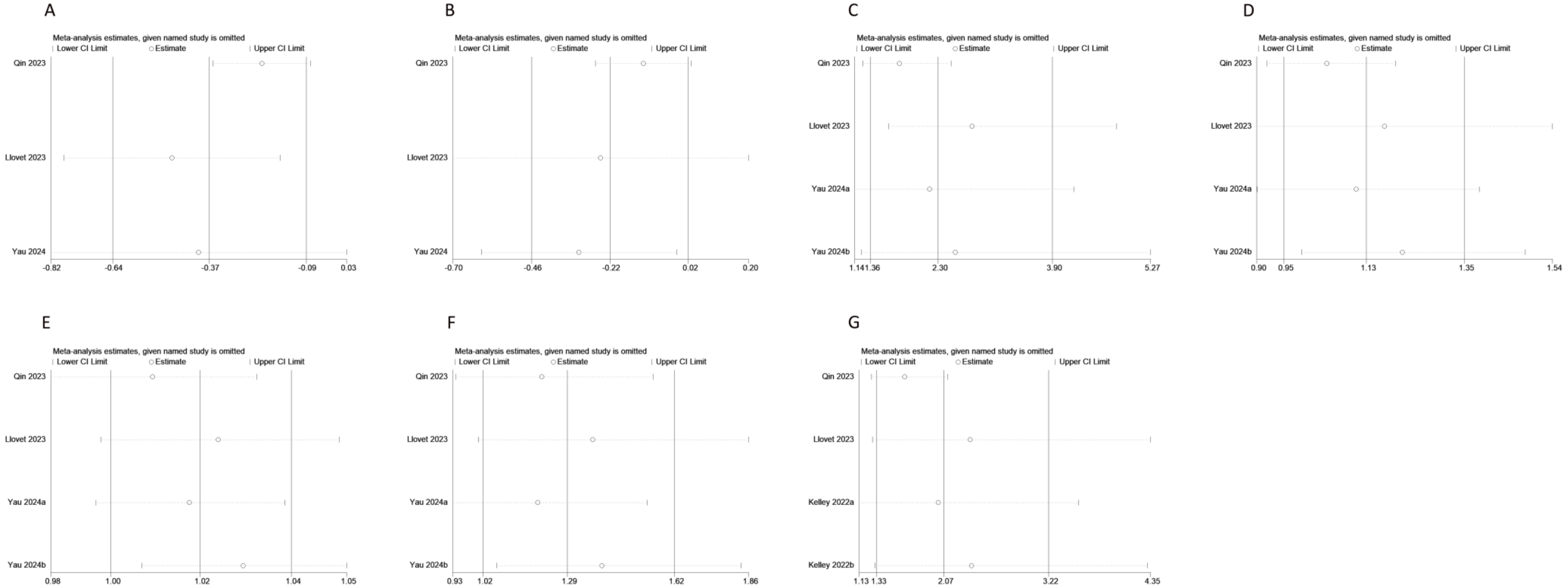

FIGURE S34 Sensitivity analysis of PD-1/PD-L1 inhibitors combined with tyrosine kinase inhibitors for hepatocellular carcinoma. (A) Progression-free survival; (B) Overall survival; (C) Objective response rate; (D) Disease control rate; (E) Any grade treatment-related adverse events (TRAEs); (F) Grade ≥ 3 TRAEs; (G) Serious TRAEs.

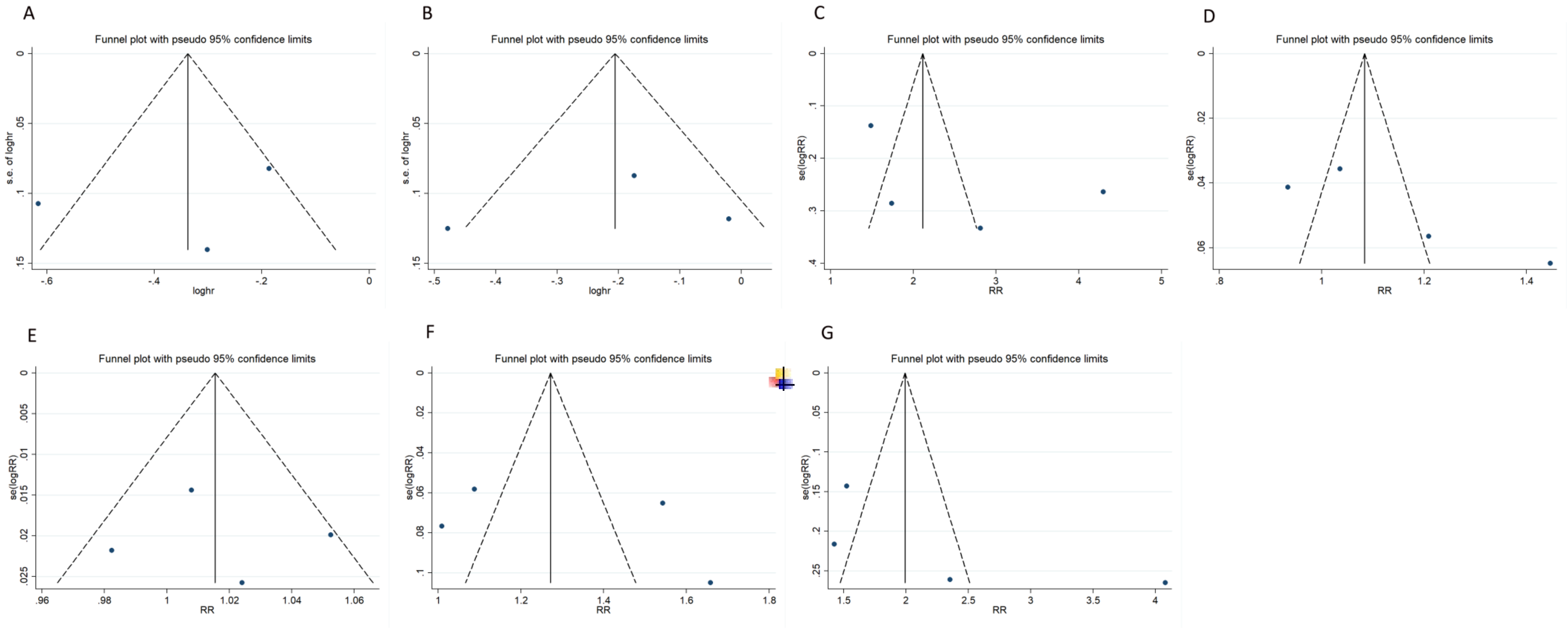

FIGURE S35 Funnel plots of PD-1/PD-L1 inhibitors combined with tyrosine kinase inhibitors for hepatocellular carcinoma. (A) Progression-free survival; (B) Overall survival; (C) Objective response rate; (D) Disease control rate; (E) Any grade treatment-related adverse events (TRAEs); (F) Grade ≥ 3 TRAEs; (G) Serious TRAEs.
